# Supplementary material for: Identification of RNA Virus–Derived RdRp Sequences in Publicly Available Transcriptomic Data Sets
Source: Mol Biol Evol. 2023 Apr 4;40(4):msad060. doi: 10.1093/molbev/msad060 (PMC10101049; doi:10.1093/molbev/msad060)
Supplement: msad060_Supplementary_Data [file msad060_supplementary_data.pdf]

Supplementary Figures

Supplementary Figure 1. Lowest pairwise identities among sequences classified to each pHMM group. All-against-all pairwise identities were calculated with BLASTP (Altschul et al., 1990; Camacho et al., 2009). For each pHMM family, the lowest pairwise identity scores for NCBI sequences (green crosses) and for NCBI and TSA sequences combined (coral circles) are shown. Families were plotted only if after discarding 100%-identical trimmed RdRp sequences there was >1 NCBI sequence left. If there was no TSA sequence left, the circle was not plotted. Numbers (using the same colours) at the bottom of the graph show the numbers of unique sequences used in the BLASTP analysis.

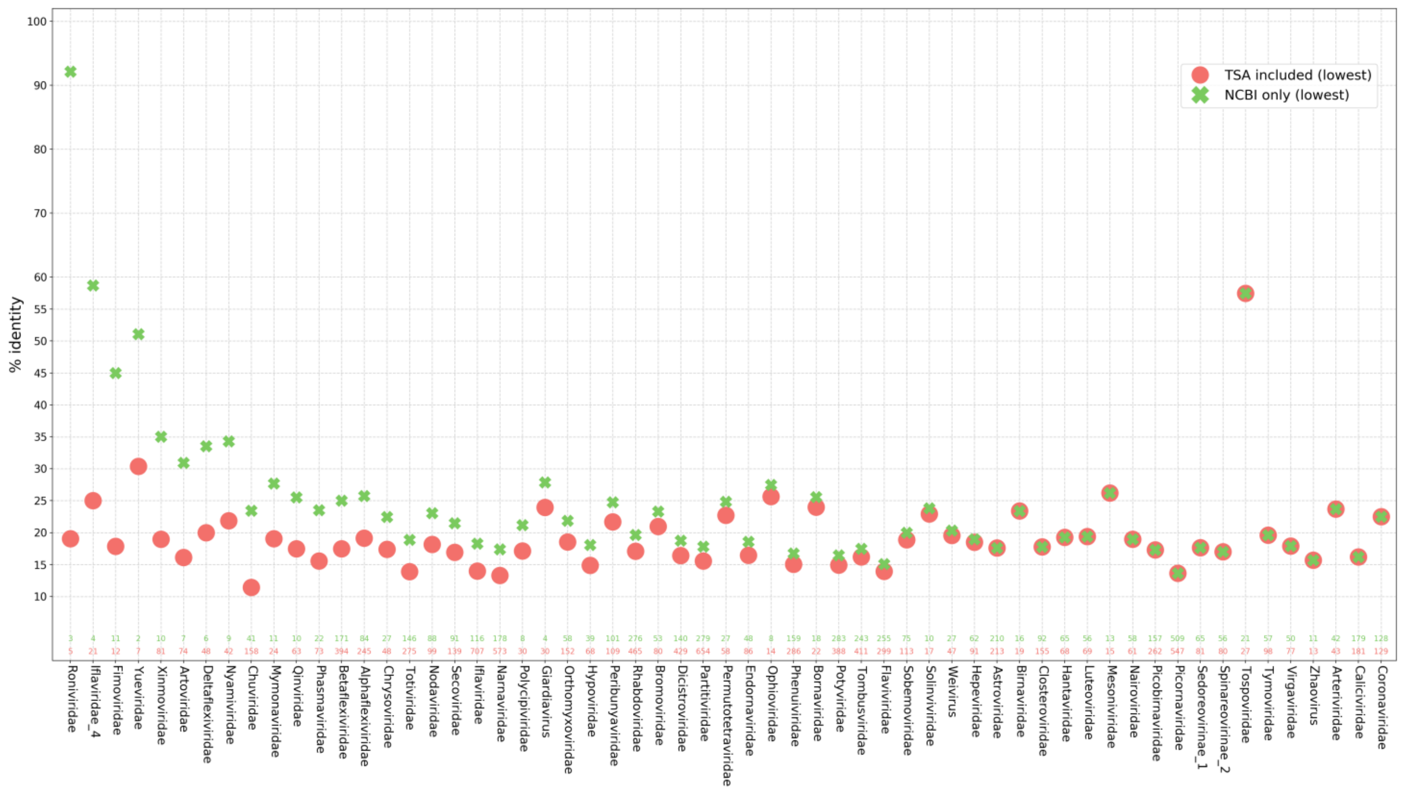

Supplementary Figure 2. Total number of classified sequences in each group of classified sequences (cluster numbers C1 to C60). Blue – nr/nt and ref sequences; pink – TSA sequences; +s stands for +ssRNA, −s for −ssRNA and ds for dsRNA virus groups.

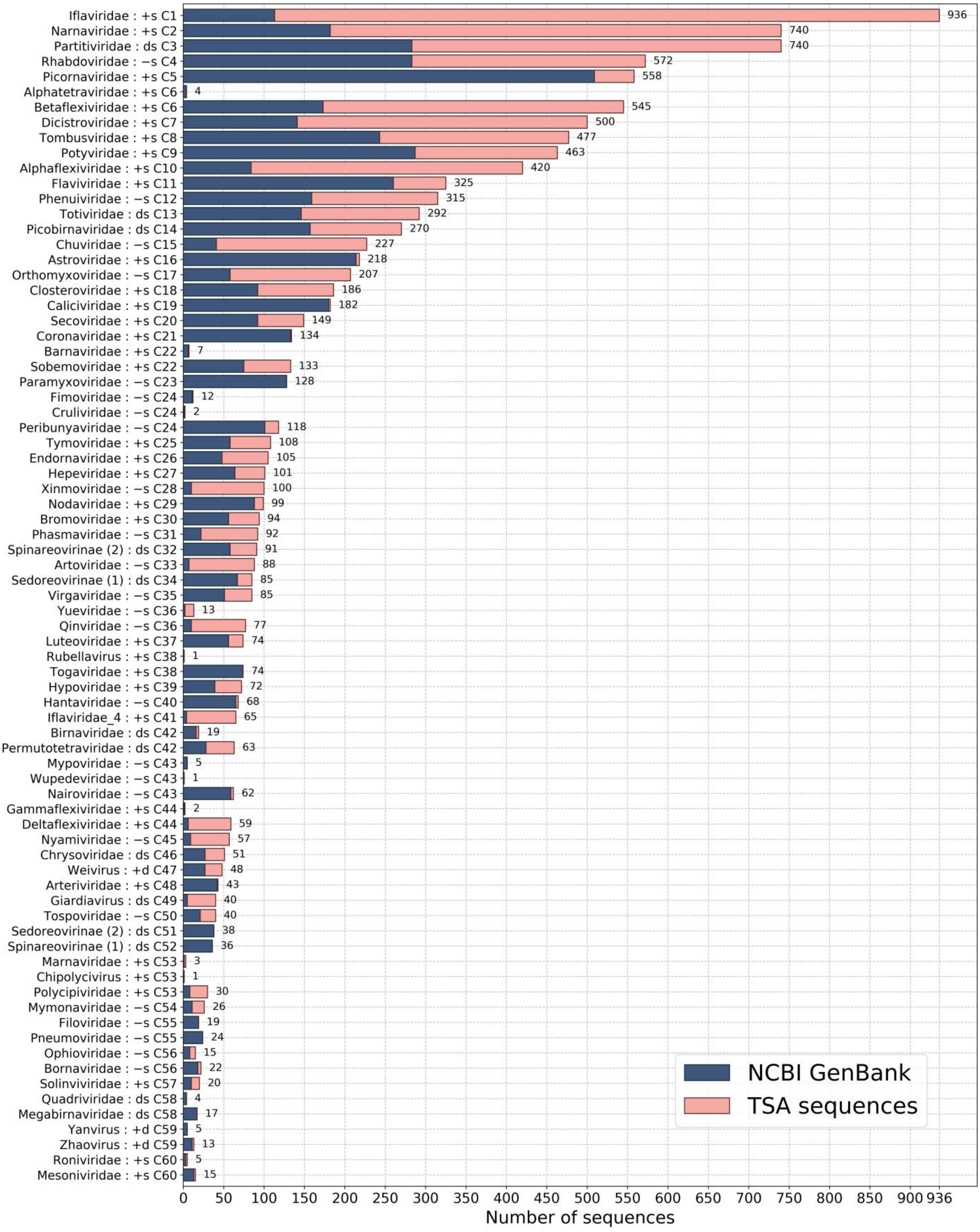

Supplementary Figure 3. Phylogenetic tree for the core RdRp sequences of chu-like viruses. Duplicate 100%-identical RdRp core sequences were removed. Blue – nr/nt sequences; pink – TSA sequences. For TSA sequences, a category icon for the putative host (i.e. the TSA target organism) is shown at right.

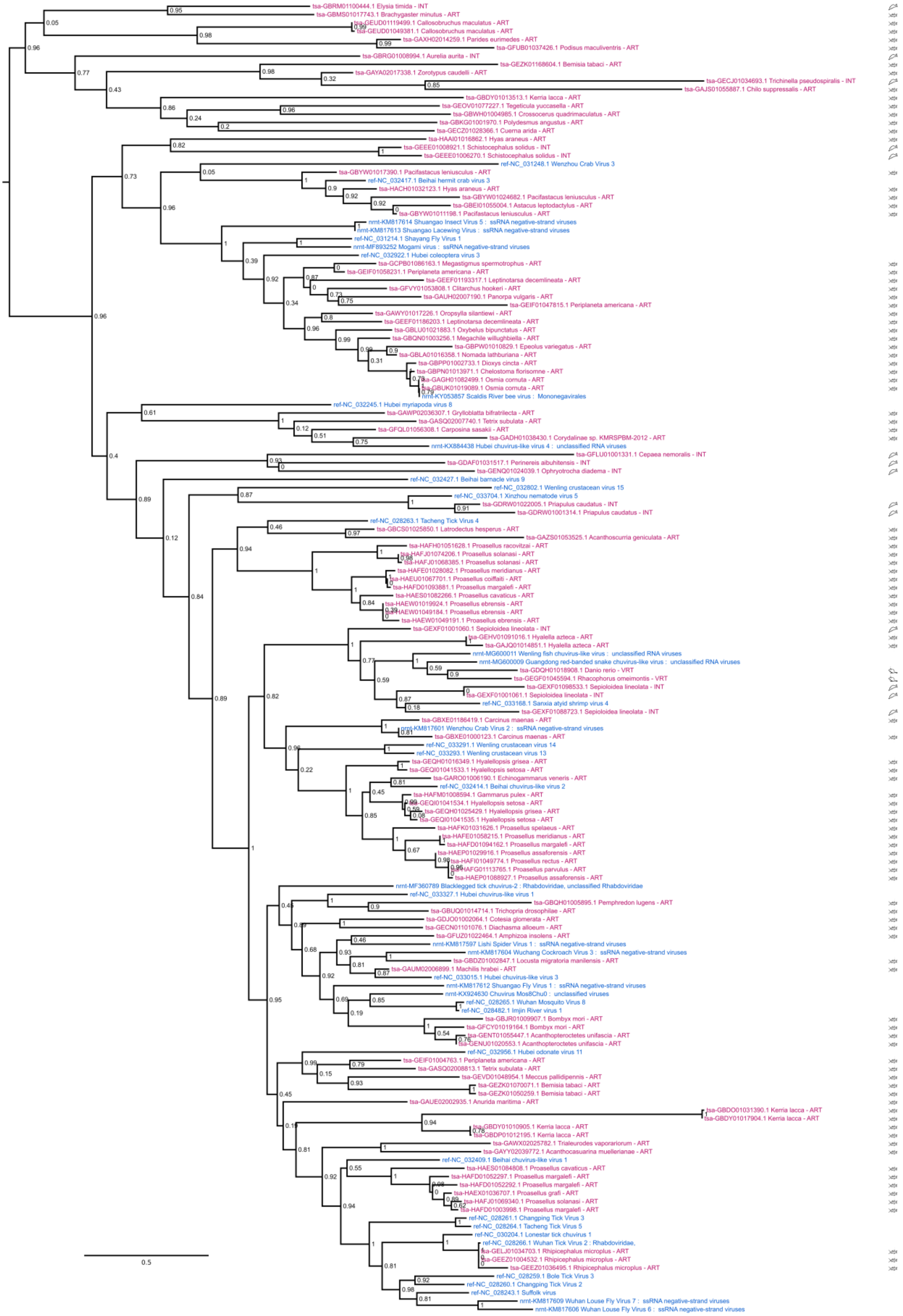

Supplementary Figure 4. Host species with highest numbers of non-identical RdRp sequences identified in TSA datasets. PLN – plant, INV – invertebrate, ENV – environmental sample.

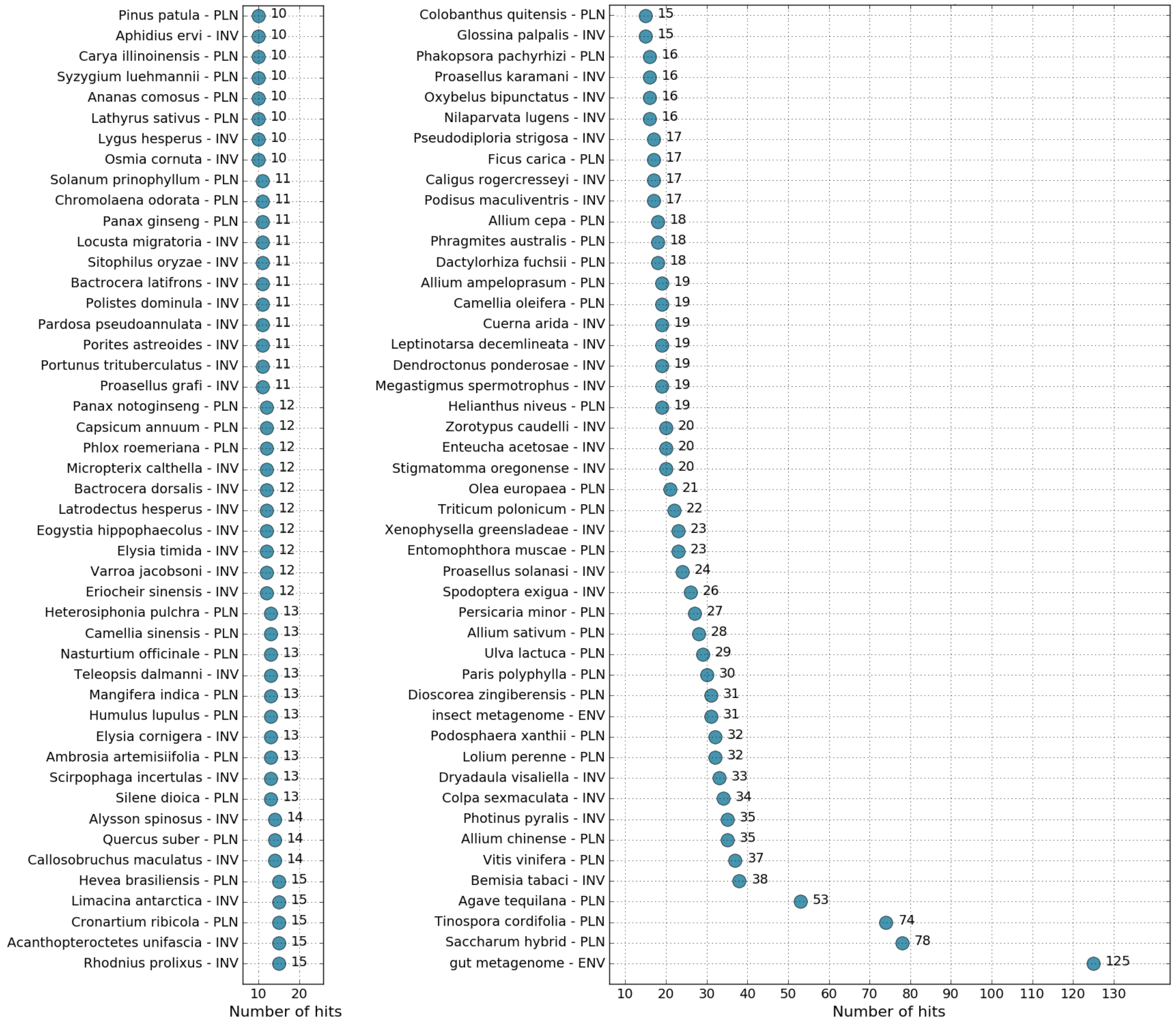

Supplementary Figure 5. Phylogenetic trees highlighting subclades of sequences where a typical GDD motif C has been replaced by an alternative sequence. **(A)** A clade of noda-like viruses with SDD. **(B)** A clade of permutotetra-like viruses with SDD. **(C)** Hypoviruses commonly have SDD but SDD-containing viruses appear to be dispersed across the hypo-like virus phylogeny. **(D)** A clade of tombus-like viruses with GDN. **(E)** A clade of chu-like viruses with GDN. See clusters 29, 42, 39, 8 and 15, respectively, in Supplementary Dataset 3 for the complete trees of each cluster from which these subtrees were extracted.

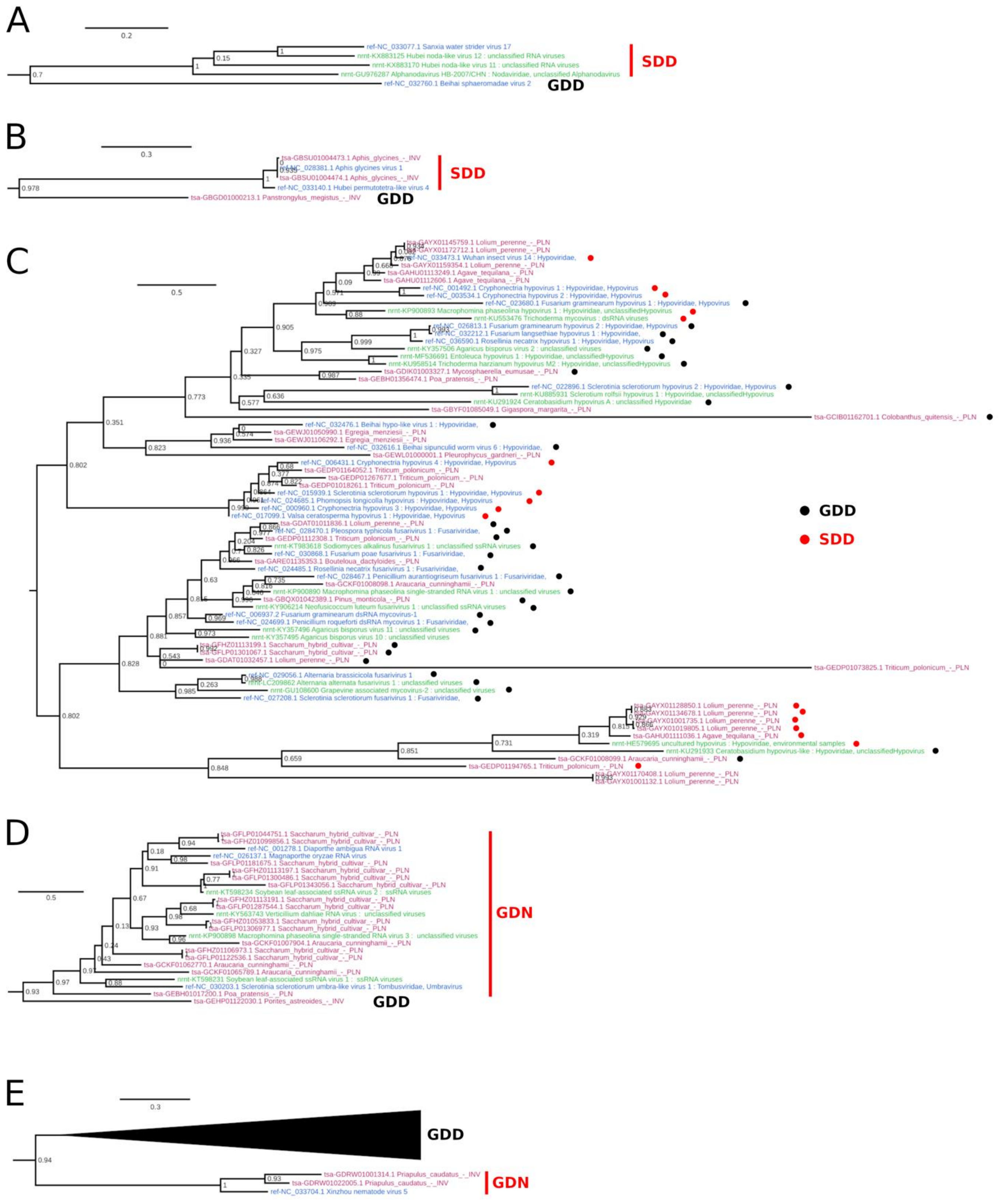

Supplementary Figure 6. Motif C amino acid and nucleotide sequences for subclades of sequences where a typical GDD motif C has been replaced by an alternative sequence. **(A)** A clade of noda-like viruses with SDD. **(B)** A clade of permutotetra-like viruses with SDD. **(C)** Hypoviruses containing SDD. **(D)** A clade of tombus-like viruses with GDN. **(E)** A clade of chu-like viruses with GDN.

(A) Noda-like sequences

Accession	Motif C	Codons (SDD)	Length	Classification
NC_033077.1	WVLGASDDRIRAN /	UCA GAU GAU	4369 nt	Sanxia water strider virus 17
KX883125.1	WVLGASDDRIRAN /	UCA GAC GAU	4727 nt	Hubei noda-like virus 12
KX883170.1	WVLGASDDRLRAN /	UCC GAU GAC	4180 nt	Hubei noda-like virus 11
GU976287.1	WVLGASDDRLRAA /	UCU GAC GAU	2952 nt	Alphanodavirus HB-2007/CHN

(B) Permutotetra-like sequences

Accession	Motif C	Codons (SDD)	Length	Classification
GBSU01004473.1	FVEAYSDDVVIYY /	AGU GAU GAC	4703 nt	Aphis glycines TSA
NC_028381.1	FVEAYSDDVVIYY /	AGU GAU GAC	4850 nt	Aphis glycines virus 1
GBSU01004474.1	FVEAYSDDVVIYY /	AGU GAU GAC	4704 nt	Aphis glycines TSA
NC_033140.1	FVEAYSDDVVIYY /	AGC GAC GAU	4772 nt	Hubei permutotetra-like virus 4

(C) Hypo-like sequences

Accession	Motif C	Codons (SDD)	Length	Classification
NC_033473.1	DIANTSDDTIWQS /	UCC GAU GAC	12473 nt	Wuhan insect virus 14
NC_001492.1	RLYNTSDDTVWWS /	AGC GAU GAC	12734 nt	Cryphonectria hypovirus 1
NC_003534.1	RFYNTSDDTVWWS /	AGU GAC GAC	12507 nt	Cryphonectria hypovirus 2
KP900893.1	IMFNTSDDLIWQS /	UCC GAC GAC	12468 nt	Macrophomina phaseolina hypovirus 1
KU553476.1	EYYNQSDDAMWLT /	UCU GAU GAU	1848 nt	Trichoderma mycovirus
NC_006431.1	KFTAFSDDNFWST /	AGU GAU GAC	9149 nt	Cryphonectria hypovirus 4
NC_015939.1	KFSSFSDDNFWST /	UCA GAU GAC	10438 nt	Sclerotinia sclerotiorum hypovirus 1
NC_024685.1	KFSSFSDDNFWST /	UCG GAU GAC	9760 nt	Phomopsis longicolla hypovirus
NC_000960.1	KFSSFSDDNFWST /	UCA GAU GAC	9799 nt	Cryphonectria hypovirus 3
NC_017099.1	KFSSFSDDNFWST /	UCG GAU GAC	9543 nt	Valsa ceratosperma hypovirus 1
GAYX01128850.1	DVANTSDDTIWQS /	UCA GAU GAC	663 nt	Lolium perenne TSA
GAYX01134678.1	DVANTSDDTIWQS /	UCA GAU GAC	469 nt	Lolium perenne TSA
GAYX01001735.1	DVANTSDDTIWQS /	UCU GAU GAU	757 nt	Lolium perenne TSA
GAYX01019805.1	DVANTSDDTIWQS /	UCU GAU GAU	705 nt	Lolium perenne TSA
GAHU01111036.1	TIANTSDDTIWQS /	UCU GAU GAC	2304 nt	Agave tequilana TSA
HE579695.1	DIANTSDDTIWQT /	UCG GAU GAC	1255 nt	uncultured hypovirus
GEDP01194765.1	KFSSFSDDNFWST /	UCC GAU GAC	611 nt	Triticum polonicum TSA

(D) Tombus-like sequences

Accession	Motif C	Codons (GDN)	Length	Classification
NC_001278.1	DFLCDGDNALLFV /	GGG GAC AAU	4113 nt	Diaporthe ambigua RNA virus 1
NC_026137.1	DLLVDGDNALVFM /	GGC GAC AAU	3246 nt	Magnaporthe oryzae RNA virus
GFLP01181675.1	DMLIDGDNALVFL /	GGU GAC AAC	3846 nt	Saccharum hybrid cultivar TSA
GFHZ01113197.1	DVLVDGDNVLVFL /	GGU GAC AAU	699 nt	Saccharum hybrid cultivar TSA
GFLP01300486.1	DVLVDGDNVLVFL /	GGU GAC AAU	699 nt	Saccharum hybrid cultivar TSA
KT598234.1	DVLVDGDNVLVFL /	GGU GAC AAU	3519 nt	Soybean leaf-associated ssRNA virus 2
GFHZ01113191.1	DTLVDGDNALVFV /	GGA GAC AAU	1137 nt	Saccharum hybrid cultivar TSA
GFLP01287544.1	DTLVDGDNALVFV /	GGA GAC AAU	1137 nt	Saccharum hybrid cultivar TSA
KY563743.1	DLLVDGDNALVFL /	GGG GAC AAC	2631 nt	Verticillium dahliae RNA virus
KP900898.2	DCLVDGDNALVFL /	GGU GAC AAU	1893 nt	Macrophomina phaseolina ssRNA virus 3
GFHZ01106973.1	DLLVDGDNALVFL /	GGU GAC AAU	975 nt	Saccharum hybrid cultivar TSA
GFLP01122536.1	DLLVDGDNALVFL /	GGU GAC AAU	975 nt	Saccharum hybrid cultivar TSA
KT598231.1	TLLADGDNALVFV /	GGC GAC AAC	4188 nt	Soybean leaf-associated ssRNA virus 1
NC_030203.1	TFLADGDNALVFV /	GGC GAC AAU	4483 nt	Sclerotinia sclerotiorum umbra-like virus 1
GEBH01017200.1	DFLADGDNAVVFV /	GGA GAC AAU	2752 nt	Poa pratensis TSA

(E) Chu-like sequences

Accession	Motif C	Codons (GDN)	Length	Classification
GDRW01001314.1	HFMYIGDNFIAKV /	GGG GAC AAC	351 nt	Priapulus caudatus TSA
GDRW01022005.1	HFMDMGDNFIAKI /	GGG GAC AAC	353 nt	Priapulus caudatus TSA
NC_033704.1	YFMDMGDNFVVKL /	GGU GAU AAU	7977 nt	Xinzhou nematode virus 5

Supplementary Figure 7. Undirected network diagram linking the HMM models which had the highest and second highest scores against RdRp ORFs identified with our pipeline. All classified-group ref, nr/nt and TSA RdRp ORFs were used (Supplementary Dataset 1). Nodes with no edges are not shown. Colours represent class, shapes represent phylum and outline styles represent Baltimore classification (+ssRNA, −ssRNA or dsRNA) as indicated in the legend. Networks were created with the Python networkx package (version 2.5.1, Hagberg et al., 2008) and visualised using Cytoscape (version 3.6.1, Shannon et al., 2003) with "degree sorted circle" layout.

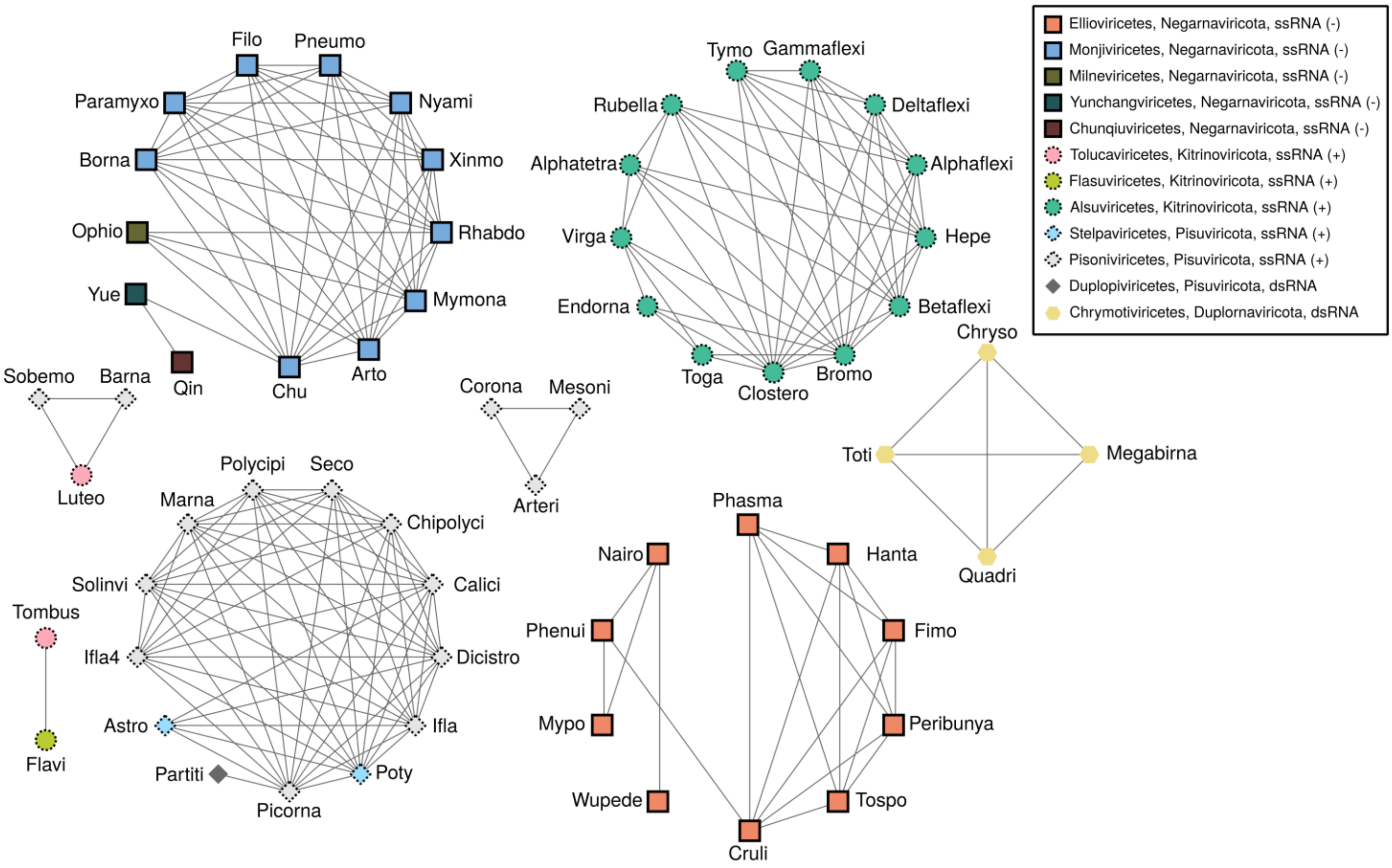

Supplementary Figure 8. Numbers of unclassified sequences. Total count (TSA, ref and nr/nt) – blue; TSA only – red. The unclassified sequences were sorted according to the best-match pHMM virus group (43 out of 77 pHMM groups being represented).

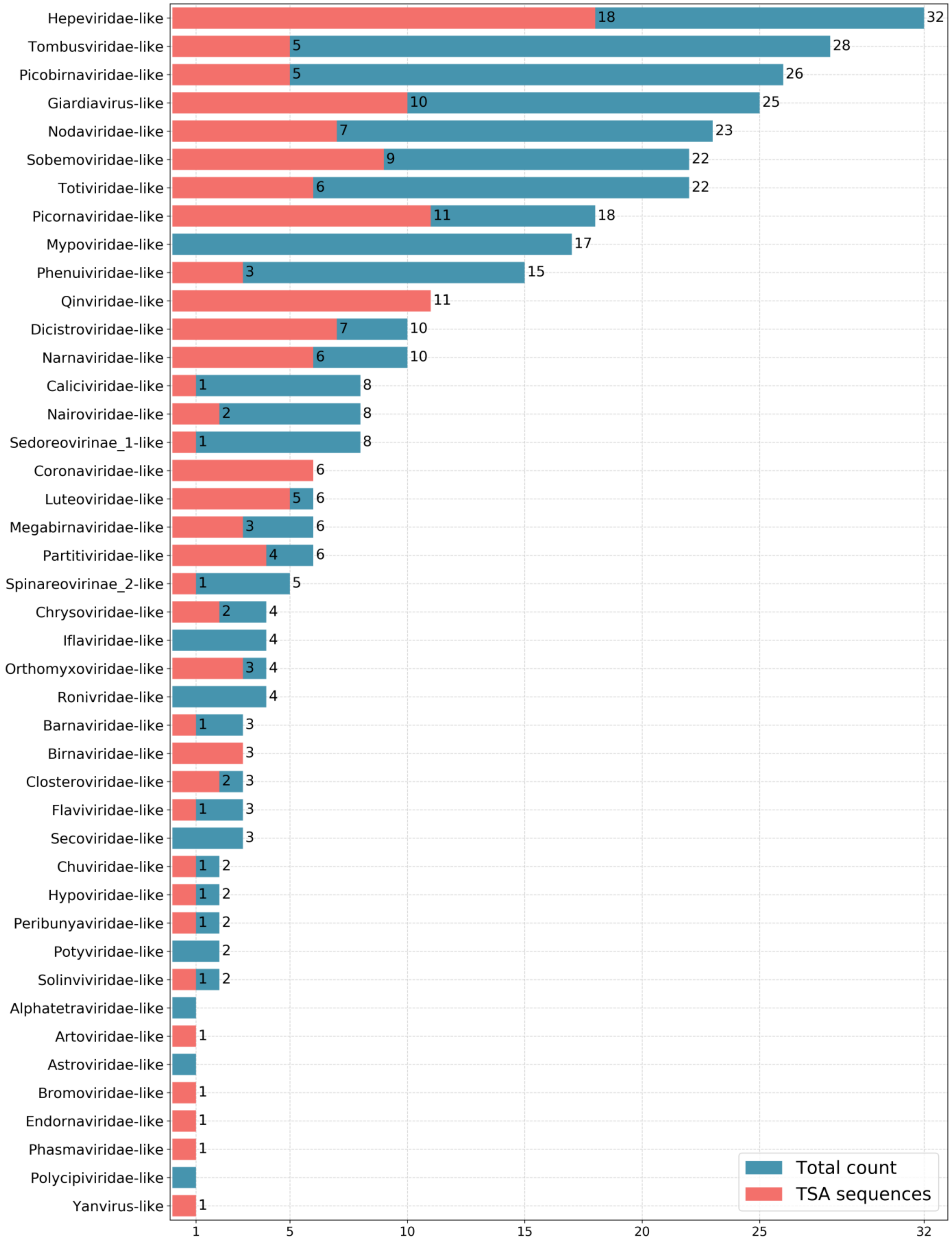

Supplementary Figure 9. Histograms of lengths of the RdRp-encoding contigs, their full RdRp-encoding ORFs, and the RdRp ORFs when trimmed to the RdRp core, for the "unclassified" group.

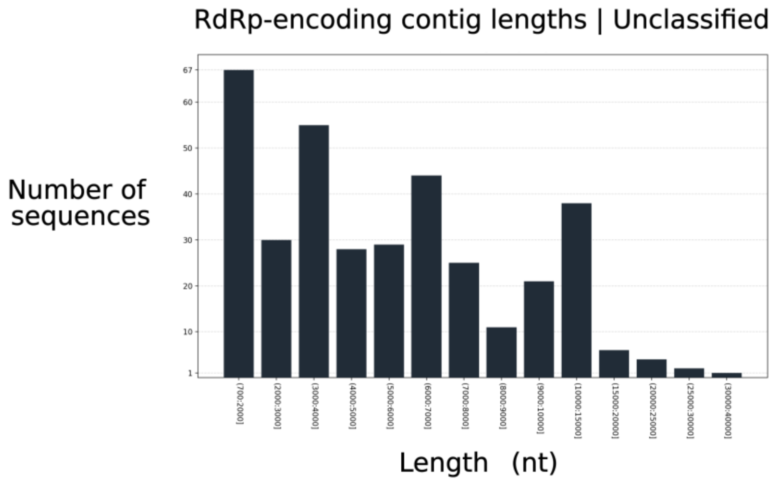

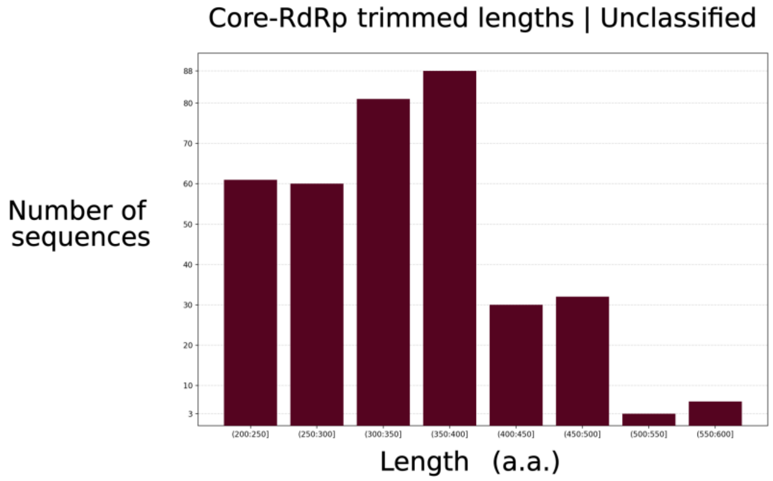

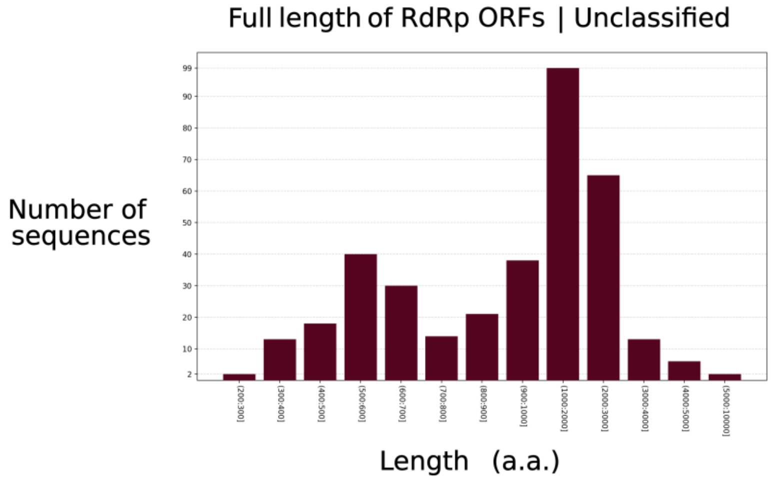

Supplementary Figure 10. Boxplots showing the results of a BLASTP analysis comparing each RdRp ORF identified here to the most similar sequence included in the input pHMMs, excluding self matches for the Genbank sequences, by percentage identity **(A)**, alignment length **(B)** and bit score **(C)**. Sequences are categorised as classified (box labelled C), ambiguous (A) or unclassified (U) based on the IDscore (HMMER bit score / HMMER alignment length) of the sequence against the best and second best matched pHMM.

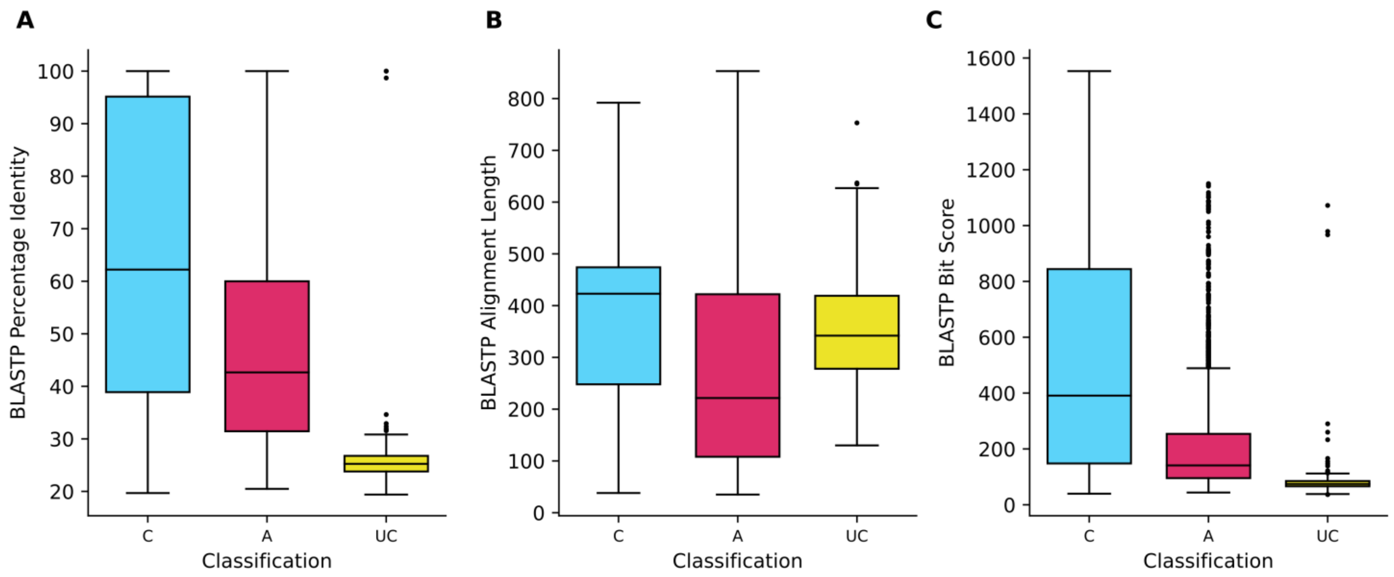

Supplementary Figure 11. PhyML (Guindon & Gascuel, 2003; Guindon et al., 2010) tree based on the core RdRp region of 80 representative sequences classified to the *Totiviridae* pHMM, besides 22 unclassified sequences with best match to the *Totiviridae* pHMM. Four major clades are annotated (see main text). A subclade containing unusually long sequences (corresponding to a previously proposed family, Fusagraviridae) is also annotated.

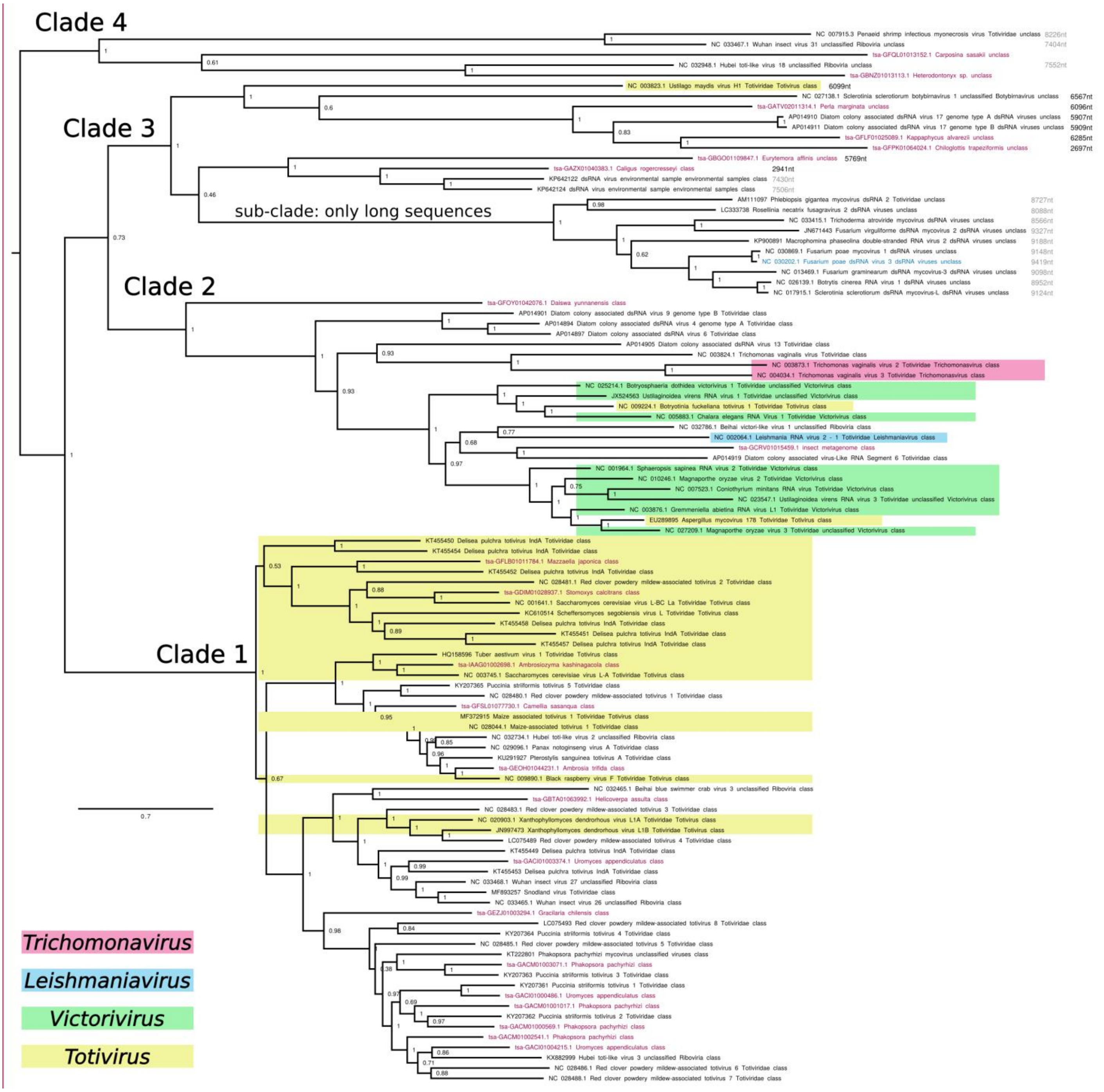

Supplementary Figure 12. PhyML tree based on the core RdRp region of sequencesclassified to the Giardiavirus pHMM, besides unclassified sequences with best match to the Giardiavirus pHMM. Red box – Giardia lamblia virus clade. Green box – clade of mainly crustacean-infecting giardia-like viruses. Note that the outlying position of GAJC01026330.1 is a result of it containing a fragmented RdRp ORF (a likely EVE sequence).

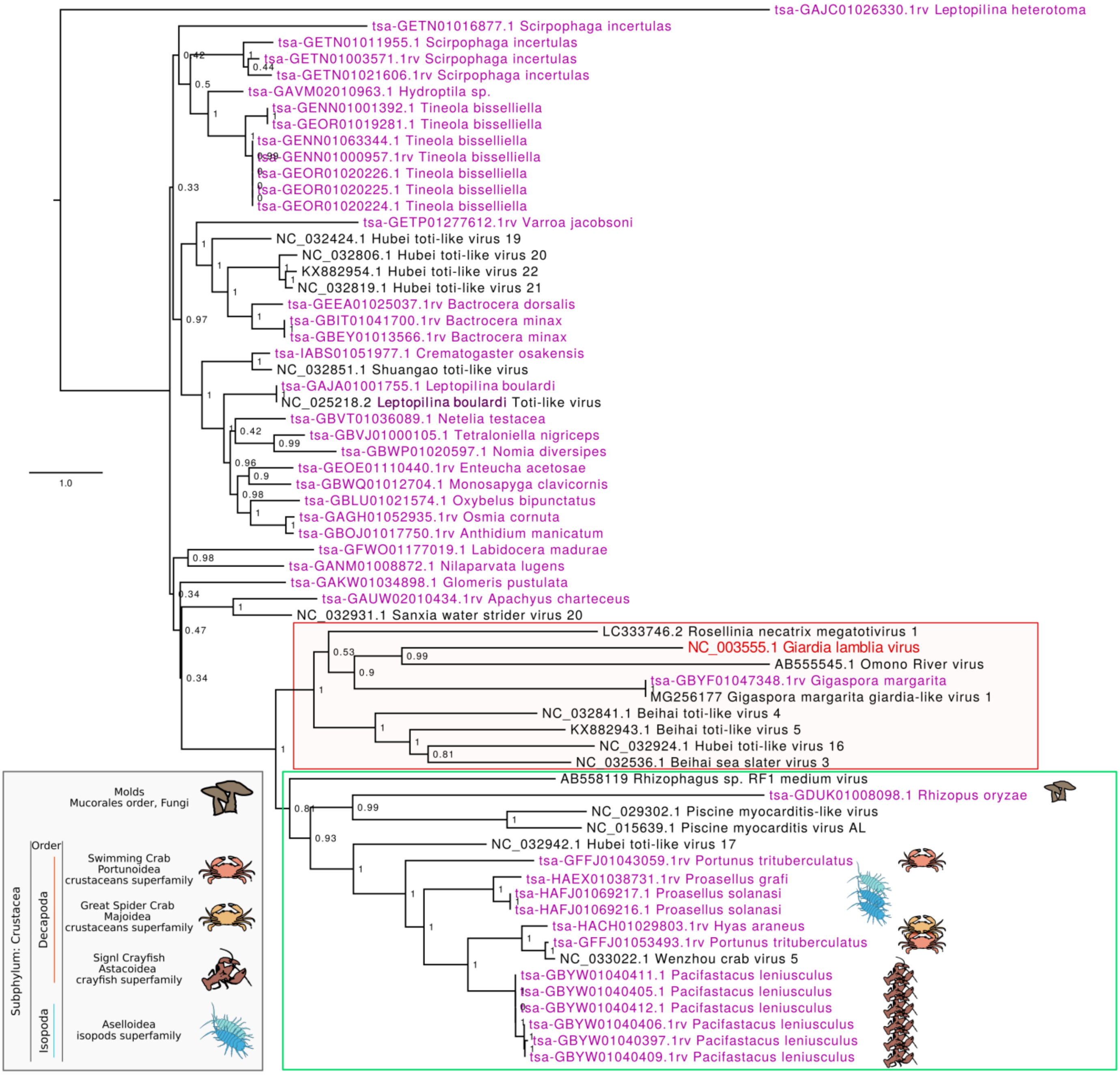

Supplementary Figure 13. Sequences in the Asellidae-associated clade of orthomyxovirus-like sequences. **(A)** Pairwise nucleotide identities for Asellidae-associated PB1-encoding contigs. Identities were calculated with Biopython pairwise2 (globalms, match score 1, nonidentical score 0, opening gap −0.1, extending gap −0.01) (Cock et al., 2009). Alignment identity scores were normalized by the length of the shorter sequence in each sequence pair. **(B)**. Coverage and identity values for BLASTX comparisons between Asellidae-associated PB1-encoding contigs and various *Orthomyxoviridae* PB1 sequences (see Supplementary Table 3 for BLASTX E-values).

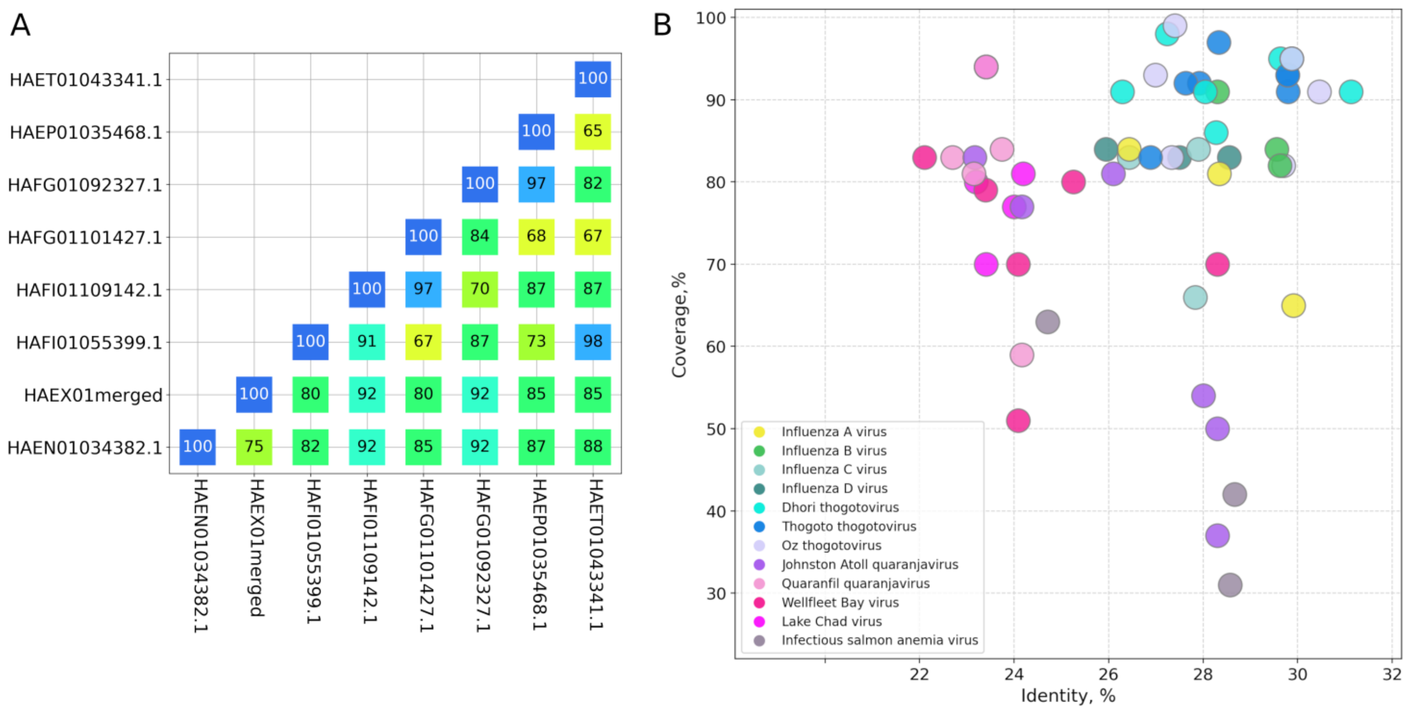

Supplementary Figure 14. Phylogenetic tree of order *Mononegavirales* L protein sequences showing the placement of the GEZL01-derived rhabdo-like virus (red arrow). NCBI RefSeq *Mononegavirales* L protein sequences were downloaded and similar sequences were discarded using CD-HIT (Li & Godzik, 2006; Fu et al., 2012) with a 90% identity threshold (cdhit -c 0.9), keeping the longest sequence in each cluster. These sequences were supplemented with the GEZL01-derived rhabdo-like virus and two additional TSA sequences identified by applying TBLASTX to the GEZL01-derived sequence, querying NCBI nr/nt sequences as well as Asteraceae TSA datasets. L protein sequences were aligned with MUSCLE (Edgar, 2004), and a phylogenetic tree was inferred using PhyML with default settings. Members of different *Mononegavirales* families (labelled at right) are indicated in different colours. Sequences/groups with known splicing are indicated in red.

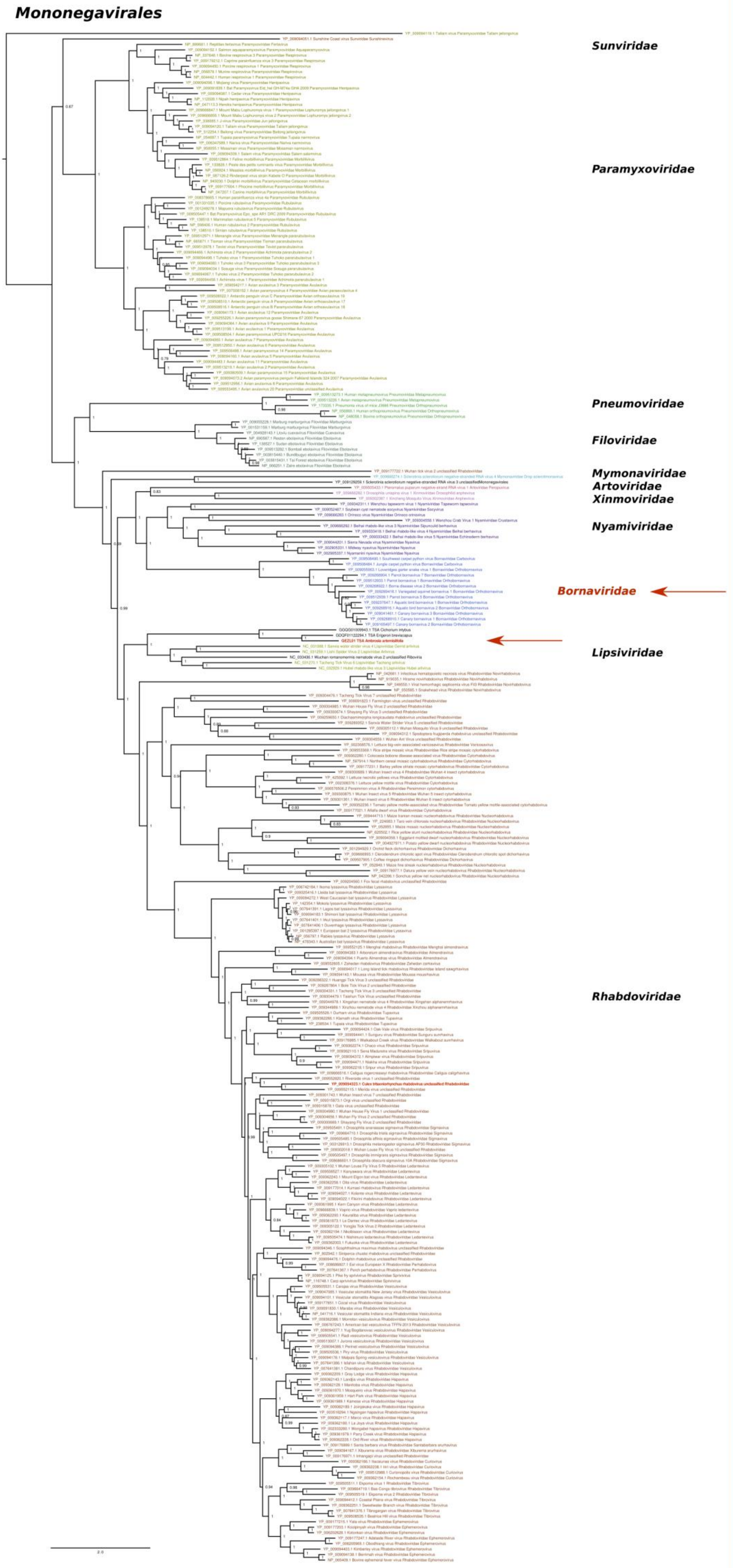
Mononegavirales
Sunviridae
Paramyxoviridae
Pneumoviridae
Filoviridae
Mymonaviridae
Artoviridae
Xinmoviridae
Nyamiviridae
Bornaviridae
Lipsiviridae
Rhabdoviridae
2.0

Supplementary Figure 15. Splicing in the GEZL01-derived sequence. **(A)** Relative nucleotide frequencies from 998 Arabidopsis thaliana splice sites (data from Brown et al., 1996); sequence logo produced with WebLogo (Crooks et al., 2004). **(B)** Section of the GEZL01-derived sequence from the third transcription stop-start sequence to the end of the merged contig. The locations of the putative L ORF and introns supported by Hisat2 spliced read mapping are annotated. Removal of introns 1–3 (blue highlight) is required to express an intact L protein (yellow highlight). Splicing of the alternative introns 1 or 4 (underlined) disrupts the L protein ORF leading to truncation, whereas splicing of intron 4 (red) removes 70 a.a. from the L protein.

A

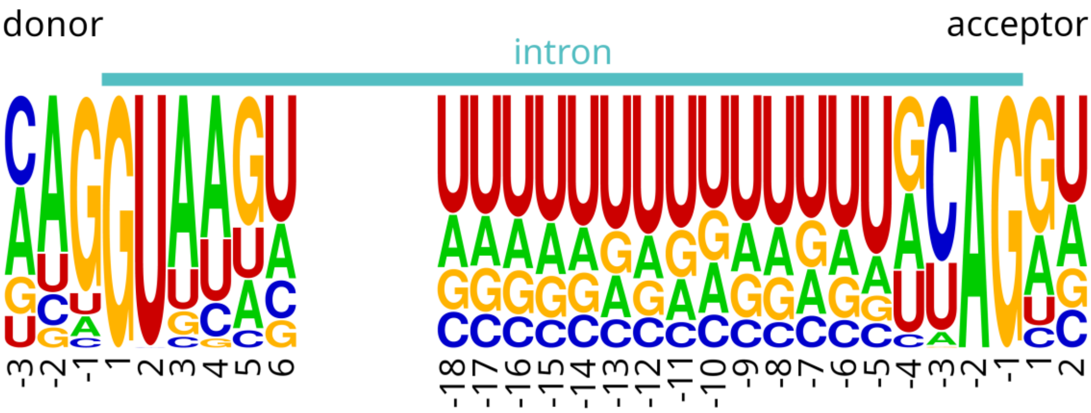

B

```
xxx Transcription stop-start sequence
xxx Introns 1, 2 and 3
AUG First AUG codon on transcript
xxx L protein coding sequence
UAA L ORF stop codon

Putatively functional introns:
Intron 1: CAG|GUAUUUCA ... CAAAUGCCUGCAG|GUC  132 nt
Intron 2: CAG|GUGAGUGA ... UAUUUUUACUUAG|GUA   98 nt
Intron 3: CUG|GUAAGUCU ... CACACUUAUGCAG|GAC  131 nt

Putatively spurious/poison introns:
Alternative intron 1: AAG|GUCAAGUU ... CAAAUGCCUGCAG|GUC  88 nt
Intron 4:             GAG|GUCUCGAU ... CGUUAUCGUGCAG|GCU 210 nt
Alternative intron 4: GAG|GUGUAUGU ... CGUUAUCGUGCAG|GCU 110 nt

ACUUUAAAAAAGUGAAAGGCAUCACGGUUUCAAUGGCUUUCUUAUACGAUUCAGACGAUGAACUCAACAACUACUCCUCUAGACAAUUGGCU
AGAUUUCCUGCAUCUCAUCACAGCUCACCGCUUACUUAUUACCCGCAAAGACAUGUAAUCAAUAUGUUGAAUGGCAGCUAUAGUGGAAAUCG
CUCUACCGUCCUCGUGGCGAUGAGGCAAACUGUGAUAAAUCUCAACAAUCAAUUAGGAAGGGGCUACACCGCUUCGGAUGAUCCUUCAAUUA
UUUAUUAUCAUUUCUUAAGCAGGUAUUUCAAAUAAAUGCAUGUAACUCUAGAUAUACUUCACAAAGGUCAAGUUCAUAUUAUCGGAUGUGGU
GAAUAGCUGAUGGUGUUUCAAUAUAAAAAUGAUUUAAUUUCUGUUUCCACAAAUGCCUGCAGGUCCCAUAUAGAAGGGAUGCUUGAUGACCA
AACAAUUCCUUAUCAACACACUAUCGCGGGUUGCCUUUCUGUCGCUCUGAACGAAGUGUUGGCUGAUCUCAGAACAUUGACAGAGUCUCACC
CUGGUCUGCCAGAUUUAAUGAGUUCAUUCCAGAAUUCACAGACGCAAACUCAAAACAUCGCUAAUGAAUUAGCCGGCUCAAUGAGGUCUCGA
UCAUUCCUGGAAAGUAUUAGAAACAGCAUACAACAAAAUCAUAUGAGAAGGAAUGAGGGUCUAAUGAGGAUUGGGAAUGAUGGGCCUGUAGA
GGUGUAUGUGUCGAGAGAAAUAACAUAUUUUGUGACUAGAACCCCAGGAAGGGUCAUUACUAUGCUCAAUUACGAUCAAAUCCUCAUGUUGU
AUGAUACGUUAUCGUGCAGGCUGAUGACUAGACUGACCUUAAGGUUAAAUGAGACUAUUGGCAGAGACAAUUUACCAACAUGGGCGGUGAUA
AGAGAAAUUUAUCAGUGGGGGGAUGAUUUGCUGCACACCUAUGGCGUUAAAGCGUAUGCACUAAUUGGAGAGUUCGAAUCAAUAGUGACAGG
AGUUUUUAUUACUUUCCAACAGACUGACGUGCUACACUUGGGUGAAGACUUUCUGAACUCACUCUCAGAAACUGCCGAGGAGCUAGAAGCUG
AGUAUGGCAUAGAUCGCAGUCACACCGUUGAGCUGAUAACAAUACUUAGAAGUGUUGGAUCUGUAAACUGGCUGAGUGAGAUAUUUGGAUUG
AGAAAACACUGGGGUAAUCCUAUGGUCGAUGCAGCAUCUUCAGGAAUGGCAGUUCAAGAGAAAAUUGCUGAAGAAUUACCUAUCAGUGGGGC
UGCAAUGGUAAAGCUACUGGCGUCCUUUAACAGGAUGAUCGUUACCGAAUAUAUUAAUCAACAUGGGAAGUGGCCAGCGGGUUACUUCUGUU
GUGAGGAGGAGGACAAUCCACUAAGCCGGGCACACUCUGCAGGAGUGACACGACUACCAGAGACUGAUGUAGAUCAUAAGCACGAACAUUGG
GCUUUGUUUAAGUUCCUUCCUAAUUGUGUUUUAGACUCUUACCAUAAUGAUCUUUCACUGGUCGGAGAUAAGUCUGUUAGUGUGUACAGAAG
CCAUCUGGACAGUAUUUAUGUUCGGAGUCUUUGUAGGGUUCCCAUACCUGCAAACAUCCAGGAAGAUCGAAGACUUGUACUUGAGUACAUAA
GGACAAAUCACCUCUCGAUUUUAGACAUAUUUAAAAUGGUGAUGUGUGACUCUAUUCCUGGAGAAUGGUUAGCAAACAGUGCCUCCAUGAAG
```

GGGACCGAAAUGAAGCACAAGAAGGCUCGGAUAUUUGCAGUCUUGACAUAUGAGUUAAGACAAUAUUUCGCUGCAACUGAAGACAUCCUCAA
GAACAGCCUAUUCAGGUGAGUGAAGUCAUUGCAAAUGAUAUUCCAAUUAGUAGACAGGCGUCAUAUCUAGAUAUAACUAUUUUAAAAUACAA
UUGGUUACUAUUUUUACUUAGGUAUAAUCCGCAUCAAACAAUGACCCUAAGUGAAGCAGAGUUGUUAUCUCGUCUUCGAAAUGUGACCCGAC
AGUUGGAUCCAUCCGGAGAUUCUAGAUAUCUCUCCGUGAUGCACAUAGUUGACUUUUCCAAAUGGAAUCUCCGGAUGAGAUAUGCUAACACC
UGCCUGAUCUUCAAAUCUAUAGAUGAUUUCUUAGGAACUCCUGGUAAGUCUCCAAGCCUACUCUUUCAUUAUUCUUCAUUUCUUUUGUUCGA
GUUUUUAUAAAAUAAACACAUAUACAUUAAAAUGAACACUUGUUUGUCAUCACUCAGAAUAACCUUUGCCACACUUAUGCAGGACUAAUUGA
GAGAUCCCACAUGAUAUUCAGCAACAUGAUCAAUAUAGUCCAAGACGCUUUCAACCCGCCCGAGCACGGUGUAUUGAAUACAGAAUCUGAAA
CAGUGUGGACAAACCACAAUUGCGGAUGGGAAGGGCAGCGUCAAAAGGGAUGGACUGUACUGACAUCAGCCCUGUUGUCGGAUUUAGCAAGA
GUAACAGGGGUGAACUCUGUGAUUUGUGGCCAAGGAGACAAUCAAACACUCUUAGCACAAUUUGCUGUGCCUGAGCAAUAUAGAAACCCCUA
CGAAUGGAUAAACAAUGAUGCAGUGGGAGUGAGACAGGUGGUAGAUACAUAUUAUAAUAAACUAGAGGAGAUAUGUCUAGAGGUUGGGUUGA
AGGUCAAGACAGCUGAGACAUGUCGCAGUCUGUCAUUUUUUAAUUAUGGCAAAAGACUAUAUAUGAAUGGUGUUGAACUGUCCAUGACUCUU
AAAAGGAUUUGCAAGAACAUAACAGAGCCAAAUGACAGUUAUCCAACCUCUGAGUCAUUACUAAAAACCCUAGAGGGUUCAGCAUUUGCUGC
AGGUCUGUACAGCCACCCUAGUAUACUGCCGUACUUGAUAAGUCGAUUCUCUGUUGCGUUACAGUUAGAGGCGUGCUUCAAAAACAACCAAU
UAAUACAACGAACCGGGAUGGAUCUCCUCAGCGAGGCUGGUUAUACCAAUUUUUACAUGUUUCUCGGAGUACUGCUUAAAAUCCCAGCGACA
CUAGGAGGACCUCCUCAACUUUCACUUGCCGCCUAUCUCUACCGUGGUCAUCCCGAUCCUCUUUGCACAGACUUGGUCUUAUUGGUUUUGCG
AGCCGGUCAUGGUUGCUUAAGAAGCAAGAAAAUCUUGGACUGUGCAAGGUCAGGAGUAUGGUUUUCUCCGAUCUCAGAAUCUCGAGUGUCAU
UAAUAAAUGCCCCAUAUUCCCUAAAUCUGGAUAUCCCUAUCUCGUGUAGAAUCCCAUUAGAGCGGGAAAUCAAGCCUGCAUUACGUUCAAAA
UGCAGGAAUACACUGGUCAUGUCAAUGUUUCAGACCAAUAUGGAUAGGGAGGAGGUCAUGAUAAUUGAAAAACUGGGGAACACAACGCCGAU
ACAUCCAUUGAUAAUCAAUGAUAUCUUUUCUCGAACAUUGCCUGGAAGGAGGCAGGCGUUUCUGUCGGGUCUGGCCAACACCACCAGUCUUG
GGCGAUUAAUAUACGACACACAAACAACACCGAUGAUUUCUCGCAUACAGAAUCUAGAAAGGCAACAACUUCAGUCUUGGGUUGAGAGAGUU
AAAGAUGUUCUGUCAGCAGAAGUGGAAGAGAUGAUUUUAACGAGCCAUAAUUCAAUUGCGGACGAGUUAAGAAAUAAAUCAUGGGGUGCAGU
GUUGGUAGGGGUCACAGUUCCGCAUCCUCUAGAGCAGUUUCGUCUAAGCAGAUCUGAGUUCGAUGUGUGUACAGGAGAUUGUGCUGGGAGUU
GCGUGGAACAUCUUGUGUUAAUCCGACACAGUGAGAACAUAAUUGAAGCACCUGGUGUCGAUGCUUCUCAUAUCUCCGCCAGGAGAUCCAGA
GUUGGAUCGCCCUAUUUGGGGAGUACCAUAACAGAGGGGAUAAUCCAAAAACAAAUUUUAUGCAUGAACCCAGAUGAGUCUUGGAGAGAUGU
AAUUAGACUGAACACAAUAAGAAAGUGGAUCACUACACCGGGGACUGUUAUGGAUGACUUCUUGAUAAAGCUUAUUCAGUCUAGAACAGAUG
UAGACUUACAGGUUAUUGAUAUGGCGACUGGAGUUCCAAGUAGGCUGUCCUACAAUCACCGUGCCAGCCUUAUGUCGGUUGUCGCUACCUGU
GGUGUUAGCACCACUACCAACAUGCACUCUCGUUUGUAUCUGUCAUCAAACCGUAUGGGCCAGUAUUCGUAUAGUGAUCAGAAUUAUCAUAU
GCCGUUUGGACCAACGUUCCUGUGCUUCCAAGAGCUCCUAUGUAUAGGUGGGGUUUACUUGGAUGAGUUUUGGAGAGAAGAUAUAAGGGACG
UUUUUCAUAUUCAUGUUAGAGAGGCCAAUAUCCCUGUUGUAUUUGAUGGAUCUAUAGUGAUGAAUUCAGUUUCUACAUCUCAAGGUGCAUCA
AGGAAUCCUCCGCCGAUCCUCUUUGCGUCCACUGCAAACCUAGAAUUUGAUGUCUCUUCAGGAGCUGUGUCUUCGUCCAUUUGGACCCCAGU
UACAAGAGCCACCAGAAACCAUGUUAGAUUUGGGUAUUGUUUCCAAUUAAUCGGAGAUUCUUUUGUCCGAGAAACAGUGAUUCGUGAAACAA
UAACACGGGAUCGAUUGGCCGGGUACGAACCGAGAAAUAUUUCUGUCACUAUUGGUGAUGUGGGUAUGGUUGGUGUUGUAAAUCUCGUGAAA
UCACUGGCUUGGAACUUUUUAUUGUUAAGCCCAAUGGGCGUGUGGGAGAAUGAAGAUCAAGCAGGGUUCGGUGUACGAGCACAAAGUUUUAU
UUCUUCUUUUACUGCCAGGUUUUGGGAUACUUUCACAACGGCCCUAUCAGCAAACAGAAACAGACAGCAGUUAAUUCUCAGCUAUAGUAUGG
UGAGAAAUUCUCAUAUGACCAGACAGGUUUCUGGGAUACGUGGUGUUCUUCAGGCUGCCGUUGUGUCCGAAAUGUGGGUUCUGUUUAAUCAG
GUUAGAGAAGCAGUGCUAAUUUCUGGGGCUGACUCUCCGAUUCCAAUUUAUGCACCCUCCGCUCAAGCCUUGAAGUUGCUGUACUCAAAGUU
CGUUGAGCUGGUCUUGUACAUUUCUAUUUAUCAUGAGGGUGGGAAUGCUCGAGAUGCUAUAGAUUAUUGUCGAGAUUUUGCGACUAACAUUC
UUAGCACUUCUAUUGAUGACUUUAAUGUGAUGACUGUGAUUAUUAAAGAUGACUUUGAGCAGCUAAUGAUACGAUUGGGUCGUGAGGGGAAG
AAGUUUAUCAAUAUUGAGAAGUUCCCGUUGCAACAUCCAUGGAUAGAUCGUUUACAUGAACUGGCCCGUCCAGUGCCUCUGUCUGCAAUUAG
CCCCAACAUCUCAGACCCAGAACAGUACCCUCCGCUCUCACAACACACCCUAUUUAUAGUCCACCCCGGCUCAAGCUUCGUGUUAGCUGAUA
ACUGCACCUGUCCUCCGAUAGAACGUCGUAAACUGCUGCAUAGUGAAUGUUAUUUUGCUCAGAGCGGCCUUUUAAGCAAGUCACAUCAGAAG
UUUGUUGAUAUAGUCUGGUCUCUGAGGCUCGAUUGUGAGGGUGUGGCCUGUCUUGGAUCCAGCACUGGGUCAGAAGCCUAUGCGUUUGCAAG
UAUGCUAUCAUGUAGGAAGGUGUAUGUUAAUUGUUUGCACCUCAUUGGUGAUUUCUCCCCUCACAUGGCUGGUUCUUAUAUCCCUGCUGCCU
UUGUAACUGGUUCUUUGUCUCACAAACUAGUCAAUCCUUCUGCUGCAGCAGUUGCGAGUUCAGAUCUUUUAUUGACCUCUACGGUAAACGCG
AUAUCCAGCCAUCUUCAAGGCACUGUUAUAACAGGUCUGACCUGUGACGCAGAGCUCCCUCAUGGUUCUGAAAUUGAUGGUGUGCAAAUGCU
GUUGUUUAGACUCAUACAAUUAGUUCAGAGGUUAACUGAGACACAAUGGGUGAUUUGCAAAGUCCCAGCUGUUUGCCCUCACGUUGUGAAGA
AGUUCUGUGCUCAGCUUGCUCAGGGGUUUCAACAGAUAAGGGCUCUGCCUUCAUUAUUCUCAAGACCCGGUGUUCUUCUUGUGUAUCUGGUA
GCUACCCAACGGAAUAGCAGCGUCUCUCCUAUGCAUCCAGUCACAUCCAUUCCGUCAGUUAUCAAUGAAAAUCAAGAUCUCGGGGAGACUCU
AAGCGUUAAUUAUAUGUCUCAGAUAUCUAGAUUGUUUCAGAUUUCCCGAGAAGAGCGAGUGCUUAGAAAUAUGACCAACGUGACUGGAGAAU
CUAGUAUUCGGUUGGCAUGCCAUAACUAUAUCCCGGGAUGGGGGCAUUCUGCGUCAAAUUCUUUAAAUCGAGAAGAGGUACGACGAUCGAUU
GAACUCCUCAAAACAGUUCUGUUGCGCUUGGCAAGUGAUUAUGUGCAAGUGUUCUUCCGCACCCAUGAGGGACUGGCAAUGCCGCGAUCUUC
UAUCCUACCUCUAGGUCAAGGAGUACUGCAUCAGCGUUUGAAUAAGAUUUACAUGAUGGUGAAUAGGUGUGAGCUGUUAUUGCUGUUGACCA
GAGGAGAAACACUCCCAAUCAAUGAAACCGUUAAUAUGAUAAAGAGGUGGGUAUCUCAAUCGCAUGAGUAUCAGAUGAGAAAUCGGUCCUUU
UUACUCGAUCUGGACGUGUCUGAUUUCACAACUAAUGUGUCUCGCUACUUGUUUGAAAUACUGGGGGGAAUCUACAAAGAAGCCUUUGCCGA
UGUUGAAGGGGCACCGGCCUUGAUCCUUCAUCAUCGUAUUGACAAUGGGAGACAGUUAGAGCGAAUAGAAGAUCUGUUUGCCCAGCUACCAA
CUCGUGGGUCCCGACCAAGAGCCAACAAUUCUUGGAGAUGGAACAGACGAUCUGAGGUCCUAUGCUGGAUACUUGCUCAUCGAUUAAAUCAA
GGCCCCAUCCAAGUGAUAACAUCAAUCAGACAUGUAUUAGAUGAAUUACAUUCAAUACAUCACUGUUCUGGAAUUCAAAUAUUCGGAGAAGG
GCAGUCUAGCGCUGUUUUUCUUAUUGCGCGUGCUGCUGACGAAUUGUUGCGUGCAACCAUGCUUAAUGCAACUUCAAUUUAUGUUGUGUGUG
AUUUAAGACUAUGCCCAAUUUUGAGCCAAUAUUUAACCAUUCAGUACUAUUUGAAAGCACCUUGGAGUGGGAUUGUGCCGGAAAUCGUGUUC
GCAAUAUGUUCCCCAUCCAGUGGCGCUGGAAUGUCUGAAGUUGUAUAUCCAGAUACUGAGCACCUCGUUAAUAAGUAUGUUGAAAAUCUCAU
AACAUUUUCUGAUUGUCAAUGUUGGGACUGUGCUAUAGAACAGAGACUAUGUGAAGAAGAAGGGCUAACUCAUAUGGAGUUAGAUAGGAUCU
GUUCAUUAAGGGAGAUUGAAUCGGUUAAGGACUUGCGACAUCAGUUGUAAGUUUAAAACGCAGAGUCAGGGUUAUGGGAGGUCUUGGAUCAC
UUAGUUUAAUUAAUUGAAGUUUUAACUAAUAAAAAGCGAAGAUAACAAUGAUAUCUAACACUAAUAUUAAGCACCUAGUUCCUUAAUGGCAA
CAGUAGUUGCUGGGAAACAAAACUUUUCAAUUUCAACAUCGGAGUGAGACAGGGAACGUCUGUUACAAACAACCAAUAUAUGACCACAUACA
AGAAAUAAAUCGAACUUCUUAAACAUAACGGAGUCCGUUUUUAAGGGAGAAGAAAGAUAAACAAGAAAGGAAAAAGGAAAAGGAAUUGGUAC
AUAGAAAGAGGAAAUUUGUCAUUUUUUAUGUUUUAAUUAGAAUGUUUCAGUCGAACAUAGACAAAUCUCUCUUUUUGUCUAUGUUCCCCAAU
UUCAACAUCGG

Supplementary Figure 16. Translation of the predicted L ORF of the GEZL01-derived sequence. Only introns 1–3 were removed. The three amino acids that are encoded by a codon spanning one of the exon-exon junctions are highlighted in yellow.

```
MAFLYDSDDELNNYSSRQLARFPASHHSSPLTYYPQRHVINMLNGSYSGNRSTVLVAMRQTVINLNNQLGRGYTASDDPSIIYYHFL
SRSHIEGMLDDQTIPYQHTIAGCLSVALNEVLADLRTLTESHPGLPDLMSSFQNSQTQTQNIANELAGSMRSRSFLESIRNSIQQNH
MRRNEGLMRIGNDGPVEVYVSREITYFVTRTPGRVITMLNYDQILMLYDTLSCRLMTRLTLRLNETIGRDNLPTWAVIREIYQWGDD
LLHTYGVKAYALIGEFESIVTGVFITFQQTDVLHLGEDFLNSLSETAEELEAEYGIDRSHTVELITILRSVGSVNWLSEIFGLRKHW
GNPMVDAASSGMAVQEKIAEELPISGAAMVKLLASFNRMIVTEYINQHGKWPAGYFCCEEEDNPLSRAHSAGVTRLPETDVDHKHEH
WALFKFLPNCVLDSYHNDLSLVGDKSVSVYRSHLDSIYVRSLCRVPIPANIQEDRRLVLEYIRTNHLSILDIFKMVMCDSIPGEWLA
NSASMKGTEMKHKKARIFAVLTYELRQYFAATEDILKNSLFRYNPHQTMTLSEAELLSRLRNVTRQLDPSGDSRYLSVMHIVDFSKW
NLRMRYANTCLIFKSIDDFLGTPGLIERSHMIFSNMINIVQDAFNPPEHGVLNTESETVWTNHNCGWEGQRQKGWTVLTSALLSDLA
RVTGVNSVICGQGDNQTLLAQFAVPEQYRNPYEWINNDAVGVRQVVDTYYNKLEEICLEVGLKVKTAETCRSLSFFNYGKRLYMNGV
ELSMTLKRICKNITEPNDSYPTSESLLKTLEGSAFAAGLYSHPSILPYLISRFSVALQLEACFKNNQLIQRTGMDLLSEAGYTNFYM
FLGVLLKIPATLGGPPQLSLAAYLYRGHPDPLCTDLVLLVLRAGHGCLRSKKILDCARSGVWFSPISESRVSLINAPYSLNLDIPIS
CRIPLEREIKPALRSKCRNTLVMSMFQTNMDREEVMIIEKLGNTTPIHPLIINDIFSRTLPGRRQAFLSGLANTTSLGRLIYDTQTT
PMISRIQNLERQQLQSWVERVKDVLSAEVEEMILTSHNSIADELRNKSWGAVLVGVTVPHPLEQFRLSRSEFDVCTGDCAGSCVEHL
VLIRHSENIIEAPGVDASHISARRSRVGSPYLGSTITEGIIQKQILCMNPDESWRDVIRLNTIRKWITTPGTVMDDFLIKLIQSRTD
VDLQVIDMATGVPSRLSYNHRASLMSVVATCGVSTTTNMHSRLYLSSNRMGQYSYSDQNYHMPFGPTFLCFQELLCIGGVYLDEFWR
EDIRDVFHIHVREANIPVVFDGSIVMNSVSTSQGASRNPPPILFASTANLEFDVSSGAVSSSIWTPVTRATRNHVRFGYCFQLIGDS
FVRETVIRETITRDRLAGYEPRNISVTIGDVGMVGVVNLVKSLAWNFLLLSPMGVWENEDQAGFGVRAQSFISSFTARFWDTFTTAL
SANRNRQQLILSYSMVRNSHMTRQVSGIRGVLQAAVVSEMWVLFNQVREAVLISGADSPIPIYAPSAQALKLLYSKFVELVLYISIY
HEGGNARDAIDYCRDFATNILSTSIDDFNVMTVIIKDDFEQLMIRLGREGKKFINIEKFPLQHPWIDRLHELARPVPLSAISPNISD
PEQYPPLSQHTLFIVHPGSSFVLADNCTCPPIERRKLLHSECYFAQSGLLSKSHQKFVDIVWSLRLDCEGVACLGSSTGSEAYAFAS
MLSCRKVYVNCLHLIGDFSPHMAGSYIPAAFVTGSLSHKLVNPSAAAVASSDLLLTSTVNAISSHLQGTVITGLTCDAELPHGSEID
GVQMLLFRLIQLVQRLTETQWVICKVPAVCPHVVKKFCAQLAQGFQQIRALPSLFSRPGVLLVYLVATQRNSSVSPMHPVTSIPSVI
NENQDLGETLSVNYMSQISRLFQISREERVLRNMTNVTGESSIRLACHNYIPGWGHSASNSLNREEVRRSIELLKTVLLRLASDYVQ
VFFRTHEGLAMPRSSILPLGQGVLHQRLNKIYMMVNRCELLLLLTRGETLPINETVNMIKRWVSQSHEYQMRNRSFLLDLDVSDFTT
NVSRYLFEILGGIYKEAFADVEGAPALILHHRIDNGRQLERIEDLFAQLPTRGSRPRANNSWRWNRRSEVLCWILAHRLNQGPIQVI
TSIRHVLDELHSIHHCSGIQIFGEGQSSAVFLIARAADELLRATMLNATSIYVVCDLRLCPILSQYLTIQYYLKAPWSGIVPEIVFA
ICSPSSGAGMSEVVYPDTEHLVNKYVENLITFSDCQCWDCAIEQRLCEEEGLTHMELDRICSLREIESVKDLRHQL*
```

Supplementary Figure 17. Alignments of the predicted L protein of the GEZL01-derived sequence against related sequences from Erigeron breviscapus **(A)** and Cichorium intybus **(B)** TSA datasets. Alignments were performed using TBLASTN in 2-sequence mode. The three amino acids in the L protein of the GEZL01-derived sequence that are encoded by a codon spanning one of the intron 1–3 exon-exon junctions are highlighted in yellow. A lack of alignment gaps at these sites and continuity in the alignment elsewhere suggests that introns 1–3 are functionally utilised whereas intron 4 and the alternative introns 1 and 4 are not.

A) Tblastn match of L protein to Erigeron breviscapus TSA sequence GDQF01122294.1 (11755 nt)

E-value = 0.0   Coverage = 99%   Identities = 55.9%

```
Query  2      AFLYDSD---DELNN---YSSRQLARFPASHHSSPLTYYPQRHVINMLNGSYSGNRSTVL  55
              A+ Y  D   DE+N    Y++RQLARFPASHH SPLTY+PQRHVIN+L G+ +   + +L
Sbjct  4433   AYKYTMDRFSDEINTRTTYNARQLARFPASHHGSPLTYFPQRHVINLLTGNSTVPATPLL  4612

Query  56     VAMRQTVINLNNQLGRGYTASDDPSIIYYHFLSRSHIEGMLDDQTIPYQHTIAGCLSVAL  115
               A R  V+ LN +LG G++A+DDP+++Y HF SRS + G     ++ Y   +  CL V++
Sbjct  4613   RAFRDAVVTLNQRLGGGFSATDDPTLVYSHFFSRSRVNGTDSGESEEYSQKVRDCLRVSI  4792

Query  116    NEVLADLRTLTESHPGLPDLMSSFQNSQTQTQNIANELAGSMRSRSFLESIRNSIQQNHM  175
              +E+  D+ +L+  HP +  L+     +Q +   I+  ++ +MR+R FLE +RN+ Q NH+
Sbjct  4793   SELRKDILSLSRVHPAIEALVDIHLPTQQEISEISASVSTAMRTRLFLEKMRNTAQLNHI  4972

Query  176    RRNEGLMRIGNDGPVEVYVSREITYFVTRTPGRVITMLNYDQILMLYDTLSCRLMTRLTL  235
               RNE +  +G+DG + VY  RE+   V+R P R I +LNYDQ+LML+DT+SCRLMTR+
Sbjct  4973   DRNESMTLVGDDGSLTVYAVREMYLCVSRFPIRQIILLNYDQVLMLFDTVSCRLMTRIAT  5152

Query  236    RLNETIGRDNLPTWAVIREIYQWGDDLLHTYGVKAYALIGEFESIVTGVFITFQQTDVLH  295
               ++E +  DNLP W +I  IY+WGD+L+   G+ AYA+IGEFESIVTGVF+TFQ TDVL
Sbjct  5153   LISERMRSDNLPPWQIIERIYRWGDELILASGMDAYAVIGEFESIVTGVFVTFQDTDVLD  5332

Query  296    LGEDFLNSLSETAEELEAEYGIDRSHTVELITILRSVGSVNWLSEIFGLRKHWGNPMVDA  355
              LG  F++++S TA+E+E EYGIDR     LI+ILR + S+NWLSEIFGLRKHWGNPMVDA
Sbjct  5333   LGIGFIDAISTTAQEIEEEYGIDRQSVQNLISILREIISLNWLSEIFGLRKHWGNPMVDA  5512

Query  356    ASSGMAVQEKIAEELPISGAAMVKLLASFNRMIVTEYINQHGKWPAGYFCCEEEDNPLSR  415
              A SG AVQEKIAEELP+SG A+++LLASFNRM+V E+I+QHG+WP GYF     D P+
Sbjct  5513   AESGRAVQEKIAEELPVSGTALIRLLASFNRMVVIEFIDQHGRWPEGYFVEAGIDTPIGV  5692

Query  416    AHSAGVTRLPETDVDHKHEHWALFKFLPNCVLDSYHNDLSLVGDKSVSVYRSHLDSIYVR  475
              AH++ VTR+PETDV+H+HEHWALFKFLPNCV+DSY NDLSL+ DKSVSVY+SHLD++YVR
Sbjct  5693   AHASSVTRIPETDVEHRHEHWALFKFLPNCVVDSYQNDLSLISDKSVSVYKSHLDTVYVR  5872

Query  476    SLCRVPIPANIQEDRRLVLEYIRTNHLSILDIFKMVMCDSIPGEWLANSASMKGTEMKHK  535
              +LCRV +PA ++EDRRL+LEY+RTNHLSILDIF+++MCD+IP EWLAN++SMKGTEMKHK
Sbjct  5873   NLCRVTLPATVEEDRRLILEYLRTNHLSILDIFRVIMCDAIPDEWLANNSSMKGTEMKHK  6052

Query  536    KARIFAVLTYELRQYFAATEDILKNSLFRYNPHQTMTLSEAELLSRLRNVTRQLDPSGDS  595
              KARIFAVLTYE+RQYFAATED++KN LFRYNPHQTMT+ EAELL++LRN+TRQLDPS ++
Sbjct  6053   KARIFAVLTYEIRQYFAATEDLIKNHLFRYNPHQTMTMGEAELLAKLRNITRQLDPSAET  6232

Query  596    RYLSVMHIVDFSKWNLRMRYANTCLIFKSIDDFLGTPGLIERSHMIFSNMINIVQDAFNP  655
              R+LSVMHIVDFSKWNLRMRY NTCLIFK+IDDFLGTPGLIE++H IFSNM NIVQD+FNP
Sbjct  6233   RHLSVMHIVDFSKWNLRMRYGNTCLIFKAIDDFLGTPGLIEQTHNIFSNMTNIVQDSFNP  6412

Query  656    PEHGVLNTESETVWTNHNCGWEGQRQKGWTVLTSALLSDLARVTGVNSVICGQGDNQTLL  715
              P  GVLNTES+TVW  HNCGWEGQRQKGWT+LTSALLSDLARVTGV S ICGQGDNQTLL
Sbjct  6413   PPDGVLNTESDTVWRFHNCGWEGQRQKGWTILTSALLSDLARVTGVRSTICGQGDNQTLL  6592

Query  716    AQFAVPEQYRNPYEWINNDAVGVRQVVDTYYNKLEEICLEVGLKVKTAETCRSLSFFNYG  775
```

```
                AQF VP+ YRNP+EWI +D  GVR  ++ YY +LE+ C  VGLK+KTAETCRSL FFNYG
Sbjct  6593     AQFPVPDIYRNPHEWIQHDPDGVRDALNKYYKQLEDECTAVGLKIKTAETCRSLLFFNYG  6772

Query  776      KRLYMNGVELSMTLKRICKNITEPNDSYPTSESLLKTLEGSAFAAGLYSHPSILPYLISR  835
                KRLY+NG ELSMTLKRI KNITEPN+SYPTSESLLKTLEGSAFAAGLY HPS+LPY+ISR
Sbjct  6773     KRLYVNGGELSMTLKRISKNITEPNESYPTSESLLKTLEGSAFAAGLYCHPSLLPYIISR  6952

Query  836      FSVALQLEACFKNNQLIQRTGMDLLSEAGYTNFYMFLGVLLKIPATLGGPPQLSLAAYLY  895
                FSVALQL+ACF  NQL+      LL + G  +F  FL +L+KIPA+LGGPPQL L AYLY
Sbjct  6953     FSVALQLDACFTRNQLLGDVRSALLEQRGIRDFTGFLSILMKIPASLGGPPQLGLPAYLY  7132

Query  896      RGHPDPLCTDLVLLVLRAGHGCLRSKKILDCARSGVWFSPISESRVSLINAPYSLNLDIP  955
                RGHPDPLCTD+V++VLRA  GC RS+ +L    SG WF+  + SR SLI APYSLNLDIP
Sbjct  7133     RGHPDPLCTDMVMVVLRARSGCTRSQSMLTVLASGRWFNRAATSRSSLIAAPYSLNLDIP  7312

Query  956      ISCRIPLEREIKPALRSKCRNTLVMSMFQTNMDREEVMIIEKLGNTTPIHPLIINDIFSR  1015
                +SCRIP+EREIKPALR KCRN LV  MF  +MD  E  I++ L    P HPLI NDIFSR
Sbjct  7313     LSCRIPIEREIKPALREKCRNQLVRGMFDADMDAIESEIVDCLSQIEPFHPLIANDIFSR  7492

Query  1016     TLPGRRQAFLSGLANTTSLGRLIYDTQTTPMISRIQNLERQQLQSWVERVKDVLSAEVEE  1075
                TLPGRRQ+FLSGLANTTSLGRLI D    PMI+RI +LE  QL+SWV+RV D++ +
Sbjct  7493     TLPGRRQSFLSGLANTTSLGRLILDNHEDPMINRICDLEGFQLESWVQRVMDIVGSSQVG  7672

Query  1076     MILTSHNSIADELRNKSWGAVLVGVTVPHPLEQFRLSRSEFDVCTGDCAGSCVEHLVLIR  1135
                 +   H+ +AD LR +SWG  LVGVTVPHPLEQF +  ++ DVC   C     EHLVL R
Sbjct  7673     WVDQPHHVLADRLRTRSWGIELVGVTVPHPLEQFSIVSADHDVCGEQCDQYLEEHLVLAR  7852

Query  1136     HSENIIEAPGVDASHISARRSRVGSPYLGSTITEGIIQKQILCMNPDESWRDVIRLNTIR  1195
                HSE +I  PG DA HI++ RSR+GSPYLGSTITEGIIQKQI C+NPDE+WRDV+RLNT+R
Sbjct  7853     HSERLITLPGSDALHIASVRSRLGSPYLGSTITEGIIQKQIQCINPDETWRDVVRLNTLR  8032

Query  1196     KWITTPGTVMDDFLIKLIQSRTDVDLQVIDMATGVPSRLSYNHRASLMSVVATCGVSTTT  1255
                +WI   G+ +DD LI++I SRTDV L++I+ ATG+PSRLSYNHRASL +V+ATCGVSTTT
Sbjct  8033     RWIAREGSRLDDMLIRIISSRTDVSLEIINQATGIPSRLSYNHRASLTAVIATCGVSTTT  8212

Query  1256     NMHSRLYLSSNRMGQYSYSDQNYHMPFGPTFLCFQELLCIGGVYLDEFWREDIRDVFHIH  1315
                NMH+RLYLSSNRMGQYS+ + NYHMPFGPTFLCFQEL+CI GVY    W    R V+HIH
Sbjct  8213     NMHTRLYLSSNRMGQYSHGECNYHMPFGPTFLCFQELVCILGVYSPRSWTLSPRSVYHIH  8392

Query  1316     VREANIPVVFDGSIVMNSVSTSQGASRNPPPILFASTANLEFDVSSGAVSSSIWTPVTRA  1375
                VR  ++P+VFDGS+ ++          N P I+FA + NLEF + SG   + IW  V +A
Sbjct  8393     VRCDDVPIVFDGSLEISRTLRPNVGFGNTPAIMFARSDNLEFQIPSGVAPNLIWNAVDQA  8572

Query  1376     TRNHVRFGYCFQLIGDSFVRETVIRETITRDRLAGYEPRNISVTIGDVGMVGVVNLVKSL  1435
                    +R GY +QLI D+FV+ETV+RE++T+DR++G+EPR I VTIGD+ +VG   L+K+L
Sbjct  8573     NATEIRIGYSYQLIADNFVKETVVRESVTQDRISGFEPRMIPVTIGDISLVGAKCLIKAL  8752

Query  1436     AWNFLLLSPMGVWENEDQAGFGVRAQSFISSFTARFWDTFTTALSANRNRQQLILSYSMV  1495
                AWNFLLLSPM VW N  + GF VRA  FIS F++RFW+TFT   SA RNRQQL+  Y
Sbjct  8753     AWNFLLLSPMSVWMNRSEQGFKVRAHGFISVFSSRFWETFTVPFSAERNRQQLLRHYGGC  8932

Query  1496     RNSHMTRQVSGIRGVLQAAVVSEMWVLFNQVREAVLISGADSPIPIYAPSAQALKLLYSK  1555
                 +  MTR  SG+R +L   VVSEMW++F++++E + I+G+ +PI +YAPS  +LKLL SK
Sbjct  8933     PDVFMTRVTSGLRPILMEGVVSEMWIIFDRLKELLHIAGSTAPISLYAPSLPSLKLLLSK  9112

Query  1556     FVELVLYISIYHEGGNARDAIDYCRDFATNILSTSIDDFNVMTVIIKDDFEQLMIRLGRE  1615
                + ELV++I+I H GG   DA +Y RD ATN LS     D     ++++ DF Q++  LG E
Sbjct  9113     YHELVMFIAIAHNGGRELDAFNYSRDIATNALSGDSSDLLSCLIMLRADFSQVLEHLGLE  9292

Query  1616     GKKFINIEKFPLQHPWIDRLHELARPVPLSAISPNISDPEQYPPLSQHTLFIVHPGSSFV  1675
                      ++ +P+Q+PWID+LH ++RPV L  I    +  P  YP L++    +V
Sbjct  9293     TLHLPYVKLYPMQYPWIDKLHTISRPVELRLIDGELDMPAFYPELARCAHIVVPENPVMA  9472

Query  1676     LADNCTCPPIERRKLLHSECYFAQSGLLSKSHQKFVDIVWSLRLDCEGVACLGSSTGSEA  1735
                +   C+CP +ER+  LH+ECYF+ SG +SKSH KF+DIV SL LD  GV CLG+STGSEA
```

```
Sbjct  9473   VLRVCSCPILERQSKLHTECYFSISGKISKSHLKFMDIVLSLNLDIAGVLCLGTSTGSEA  9652

Query  1736   YAFASMLSCRKVYYVNCLHLIGDFSPHMAGSYIPAAFVTGSLSHKLVNPSAAAVASSDLLL  1795
               AF     C  VYYVNCLH + +FSPH+AGSY+PAAF TG+ S KLV PS+A +ASSDLL
Sbjct  9653   AAFCRFSQCNYVYYVNCLHPLSEFSPHIAGSYVPAAFATGAYSRKLVRPSSAVIASSDLLA  9832

Query  1796   TSTVNAISSHLQGTVITGLTCDAELPHGSEIDGVQMLLFRLIQLVQRLTETQWVICKVPA  1855
              + TV+AI  H      TG+TCDAE P         MLL  +++LV       WVICKV
Sbjct  9833   SDTVDAIRIHTSQHTFTGITCDAEFPLVDSDHSYLMLLGNILKLVADKPSIAWVICKVSY  10012

Query  1856   VCPHVVKKFCAQLAQGFQQIRALPSLFSRPGVLLVYLVATQRNSSVSPMHPVTSIPSVIN  1915
               C H + KFC+++++ F Q+R + S FSRPG+LL Y+V T R +S  P  P   +P VI
Sbjct  10013  KCVHELYKFCSEMSRAFYQVRTVWSQFSRPGILLAYIVGTHRRTSFMPERPADEVPVVIL  10192

Query  1916   ENQDLGETLSVNYMSQISRLFQ---ISREERVLRNMTNVTGESSIRLACHNYIPGWGHSA  1972
              E   L   +      ++  +F    ++R++R+       T + S+  AC   IPGWG +
Sbjct  10193  EADSLTTLVEETVRLELCPMFYEYGVTRDQRLF---VLHTADDSLYAACQKLIPGWGMNF  10363

Query  1973   SNSLNREEVRRSIELLKTVLLRLASDYVQVFFRTHEGLAMPRSSILPLGQGVLHQRLNKI  2032
                S +RE +  S+E +K  L+  A+D +++ +RTH G + PR++IL LGQG L+Q+LN +
Sbjct  10364  PISASRELIISSLEDIKLALISQANDLLRISYRTHMGRSQPRNAILQLGQGALNQKLNNV  10543

Query  2033   YMMVNRCELLLLLTRGETLPINETVNMIKRWVSQSHEYQMRNRSFLLDLDVSDFTTNVSR  2092
              Y +VNRCELLL L     L I ETV +++ WV+  HEY M +R FLLDL+ + FT  VSR
Sbjct  10544  YKLVNRCELLLRLLVDPVLSIRETVRIMRDWVATPHEYPMLSRGFLLDLNATHFTNYVSR  10723

Query  2093   YLFEILGGIYKEAFADVEGAPALILHHRIDNGRQLERIEDLFAQLPTRGSRPRANNSWRW  2152
              Y+FEILG +YK  F DV      +L HR+     +    +LF  L     R     SWRW
Sbjct  10724  YVFEILGHVYKNQFTDVNLFGDQVLFHRLTPTENIRHGGNLFRPLDLSSRRMTVKLSWRW  10903

Query  2153   NRRSEVLCWILAHRLNQGPIQVITSIRHVLDELHSIHHCSGIQIFGEGQSSAVFLIARAA  2212
               RR+E+L W+L+ RL+  PI VIT     LDE+H +     ++++ +     +   A
Sbjct  10904  LRRAELLFWMLSMRLSGSPIFVITENPIALDEIHLLGKLGDVELYTDQNEGPIACSAATV  11083

Query  2213   DELLRATMLNATSIYVVCDLRLCPILSQYLTIQYYLKAPWSGIVPEIVFAICSPSSGAGM  2272
              D ++  + L    I++     +CP +  Y  +    K PW+GI+P  V   CS  +
Sbjct  11084  DSIISLSSLTDRRIFLWTYCHMCPTIFAYFHVSQMFKCPWTGIIPNTVIVDCSSLNNEIP  11263

Query  2273   SEVVYPDTEHLVNKYVENLITFSDCQCWDCAIEQRLCEEEGLTHMELDRICSLREIESVK  2332
              S V YPD + L+N   +    F +C CWDC IE  LC + G++H  LDR+CS+  IESV+
Sbjct  11264  SSVRYPDFDSLINTAYDGGDMFCECSCWDCVIEGVLCADFGVSHSLLDRLCSMGAIESVR  11443

Query  2333   DLRHQL  2338
              D++H +
Sbjct  11444  DMQHSM  11461
```

B) Tblastn match of L protein to Cichorium intybus TSA sequence GGQG01009943.1 (7864 nt)

E-value = 0.0   Coverage = 99%   Identities = 37.3%

```
Query  18    QLARFPASHHSSPLTYYPQRHVINMLNGSYSGNRSTVLVAMRQTVINLNNQLGR--GYTA  75
             +L +FP SH S+PLTY+PQR +I      YS      +  +R  V  L     R  G
Sbjct  141   RLRKFPGSHLSAPLTYFPQRLIIQ----HYSNQTEAPIEKLRAGVRTLKFLKDRFPGCCF  308

Query  76    SDDPSIIYYHFLSRSHIEGM-LDDQTIPYQHTIAGCLSVALNEVLADLRTLTESHPGLPD  134
              +DPS IY HF  R        D  T    + ++  L     +    L+++ +  PGL
Sbjct  309   KEDPSAIYAHFFRRCTKRSTPADRNTQEVVNELSQFLFTCCQDSQMSLKSIAKQSPGLGS  488

Query  135   LMSSFQNSQTQTQNIANELAGSMRSRSFLESIRNSIQQNHMRRNE-GLMRIGNDGPVEVY  193
             L   FQ  +   + +   +  + +   F+  +R S+Q   M   E  +M   N     VY
Sbjct  489   LELKFQ--EDMIKEVIMSVLEAYKCYKFVVEVRGSVQGRVMMEAEKDIMGPFNYSGYTVY  662

Query  194   VSREITYFVTRTPGRVITMLNYDQILMLYDTLSCRLMTRLTLRLNETIGRDNLPTWAVIR  253
```

```
                E+   +  T      +LNYDQ +M+ DT+  R +T L   LN     D LP    +R
Sbjct  663   HQGEMVVCIQDTDPSETVVLNYDQWVMICDTVVSRFITLLVCELNARKPFDILPRPDFLR  842

Query  254   EIYQWGDDLLHTYGVKAYALIGEFESIVTGVFITFQQTDVLHLGEDFLNSLSETAEELEA  313
             E+Y+WGD++LH  G+ AYA+IGEFESIVT  F++F  +D L+LG DF+ S+ +TA E EA
Sbjct  843   ELYEWGDEMLHEDGIAAYAVIGEFESIVTSCFVSFADSDPLNLGADFIASVRQTALEQEA  1022

Query  314   EYGIDRSHTVELITILRSVGSVNWLSEIFGLRKHWGNPMVDAASSGMAVQEKIAEELPIS  373
             EYGI    T +L+T+LR   SVN LSE+FGLRKHWGNPMV+AA+SG AVQ+K+ E+LPIS
Sbjct  1023  EYGIRAGRTTKLMTLLRKQASVNVLSEVFGLRKHWGNPMVEAAASGRAVQDKLNEDLPIS  1202

Query  374   GAAMVKLLASFNRMIVTEYINQHGKWPAGYFCCEEEDN---PLSRAHSAGVTRLPETDVD  430
             G A+ +LLA+FNRMI   +I  H +WPA  F  E +D    PL +A  +  T +PE D +
Sbjct  1203  GVALTELLAAFNRMITLSFIKIHNRWPACEFK-ERKDGVVYPLMKAQKSNNTTIPENDPE  1379

Query  431   HKHEHWALFKFLPNCVLDSYHNDLSLVGDKSVSVYRSHLDSIYVRSLCRVPIPANIQEDR  490
             H H  WAL  F  NCV D+Y N L  + DKSVS+    LD+IY + +      A    +R
Sbjct  1380  HDHLDWALLVFHCNCVADTYENQLETLSDKSVSLPVRELDNIYYKGMIPNQKTAEGDSER  1559

Query  491   RLVLEYIRTNHLSILDIFKMVMCDSIPGEWLANSASMKGTEMKHKKARIFAVLTYELRQY  550
             RL+L++++   + ++DI K +M D  P +W ANSA+MKGTE+KH KARIFAVLTYELRQY
Sbjct  1560  RLILKFLQEEQMPVMDILKKIMTDKAPPDWFANSANMKGTEVKHLKARIFAVLTYELRQY  1739

Query  551   FAATEDILKNSLFRYNPHQTMTLSEAELLSRLRNVTRQLDPSGDSRYLSVMHIVDFSKWN  610
             FA TED++K  LF YNPHQTMT++E ELL +LR +T+Q+D    SR L+VMHIVDFSKWN
Sbjct  1740  FAVTEDMIKRHLFPYNPHQTMTMNETELLVKLRGMTKQMDVDSTSRTLNVMHIVDFSKWN  1919

Query  611   LRMRYANTCLIFKSIDDFLGTPGLIERSHMIFSNMINIVQDAFNPPEHGVLNTESETVWT  670
             LRMRY NTC +F+SID+FLG PGLIE+SH+IF +M+N+VQD +NPPE+GV NT S+T+W
Sbjct  1920  LRMRYENTCGVFQSIDNFLGFPGLIEKSHIIFEDMVNLVQDPYNPPENGVANTLSDTIWR  2099

Query  671   NHNCGWEGQRQKGWTVLTSALLSDLARVTGVNSVICGQGDNQTLLAQFAVPEQYRNPYEW  730
              H  GWEGQRQKGWT+LTSALLSDL RVTGV+S ICGQGDNQ LL QF +PEQY+N  EW
Sbjct  2100  LHTRGWEGQRQKGWTILTSALLSDLERVTGVHSEICGQGDNQVLLTQFTIPEQYKNAEEW  2279

Query  731   INNDAVGVRQVVDTYYNKLEEICLEVGLKVKTAETCRSLSFFNYGKRLYMNGVELSMTLK  790
             I ++   V+ ++D YY KLE  C  VGLKVKTAETCRSL + NYGKRL+  GVELSMTLK
Sbjct  2280  IASEPQIVKLILDDYYKKLEIKCAGVGLKVKTAETCRSLFYMNYGKRLFFCGVELSMTLK  2459

Query  791   RICKNITEPNDSYPTSESLLKTLEGSAFAAGLYSHPSILPYLISRFSVALQLEACFKNNQ  850
             R+ K +TE ND++P +E+ LKT +G+++AAGLYSHPS  P+++SRF   L L   F  N
Sbjct  2460  RLSKAMTESNDAFPLTETRLKTSQGASYAAGLYSHPSPWPFIMSRFCTMLTLYESFCFNP  2639

Query  851   LIQRTGMD-LLSEAGYTNFYMFLGVLLKIPATLGGPPQLSLAAYLYRGHPDPLCTDLVLL  909
             LI   G   LL +A  +    F  +L K+PA LGGP Q   AA+L RGHPDPLC +LV+L
Sbjct  2640  LIGDFGKKVLLQDASISG--RFFSILAKLPACLGGPSQQPFAAFLCRGHPDPLCEELVML  2813

Query  910   VLRAGHGCLRSKKILDCARSGVWFSPISES-RVSLINAPYSLNLDIPISCRIPLEREIKP  968
             +L+A  GC  SK I+ CA  G WF    +  R SLI +PYSLNL+IP S R P+   + P
Sbjct  2814  ILKARSGCAISKSIIHCAIEGSWFRDGRDGDRSSLIMSPYSLNLNIPTSARSPISHAVLP  2993

Query  969   ALRSKCRNTLVMSMFQTNMDREEVMIIEKLGNTTPIHPLIINDIFSRTLPGRRQAFLSGL  1028
             AL+S C+N+ V  +    +D  E  ++E L N TP +PLI NDI+SR++ G+RQ+F+ GL
Sbjct  2994  ALKSFCKNSSVSELLSLEVDEYEKKLLEVLSNLTPFNPLIANDIYSRSIVGQRQSFVDGL  3173

Query  1029  ANTTSLGRLIYDTQTTPMISRIQNLERQQLQSWVERVKDVLSAEVEEMILT-SHNSIADE  1087
              N TS+ RL+ D+ T P+I  I+ LE +QL  WV  VK +++ +   +     H+ +AD+
Sbjct  3174  KNATSICRLVQDSPTNPIIDEIKRLEERQLVFWVNAVKLIVANQGASIPNNLRHDQLADK  3353

Query  1088  LRNKSWGAVLVGVTVPHPLEQFRLSRSEFDVCTGDCAGSCVEHLVLIRHSENIIEAPGVD  1147
             LR KSWG  L GVTVPHPLE F L +++ D C          +LV +RHS N+   PGVD
Sbjct  3354  LREKSWGIQLTGVTVPHPLEIFSLLKADDDHCDSHEDTKDDGYLVFVRHSNNLFSPPGVD  3533

Query  1148  ASHISARRSRVGSPYLGSTITEGIIQKQILCMNPDESWRDVIRLNTIRKWITTPGTVMDD  1207
                +   RSR G PYLGS ITEGI  KQ++C NP ++W D  + N +R+WIT PG+ +D
```

```
Sbjct  3534  NGKVQMTRSRTGKPYLGSMITEGITSKQVVCKNPTKAWTDACKYNVMRQWITRPGSTLDR  3713

Query  1208  FLIKLIQSRTDVDLQVIDMATGVPSRLSYNHRASLMSVVATCGVSTTTNMHSRLYLSSNR  1267
              L  LI+SRTD D++ + + TG PSR+SY HRA   +    C ++ T+NM+SR+Y SSNR
Sbjct  3714  LLRDLIKSRTDADIEFLCLVTGRPSRISYVHRADFTATKKVCALTATSNMYSRIYTSSNR  3893

Query  1268  MGQYSYSDQNYHMPFGPTFLCFQELLCIGGVYLDE-FWREDIRDVFHIHVREANIPVVFD  1326
             MG+YS SD NYH+P+GP +L F  LLC+  V   E  W       ++H HV   +IP V+D
Sbjct  3894  MGKYSTSDVNYHLPYGPAYLTFSYLLCLSEVLTPESSWSRSDHQIYHAHVVVDDIPTVYD  4073

Query  1327  GSIVMNSVSTSQGASRNPPPILFASTANLEFDVSSGAVSSSIWTPVTRATRNHVRFGYCF  1386
             G I +++   S      PP +L++S   +EFD  +    +  +    +A+    +  Y
Sbjct  4074  GEISVDAPKNSFRYPVKPPKMLYSSVKAMEFDDLTVNSDTVTFKDFNKASIRQCQMAYSN  4253

Query  1387  QLIGDSFVRETVIRETITRDRLAGYEPRNISVTIGDVGMVGVVNLVKSLAWNFLLLSPMG  1446
              L+GDSF+RE V +E +        ++ R+I ++IGD+ + G+   VK+LA N  L+
Sbjct  4254  LLLGDSFIREIVKKELMEASITDFFQKRDIMISIGDLKLTGISGFVKALAQNMCLVMGKH  4433

Query  1447  VWENEDQAGFGVRAQSFISSFTARFWDTFTTALSANRNRQQLILSYSMVRNSHMT---RQ  1503
               +  +++ F        ++ F+  FW +F TA++     ++L   YS+   S  +
Sbjct  4434  RAQIANKSCFIYSMVDLMNGFSRGFWSSFCTAMAKPELIEKL---YSLTEGSGRSYDGAN  4604

Query  1504  VSGIRGVLQAAVVSEMWVLFNQVREAV--LISGADSPIPIYAPSAQALKLLYSKFVELVL  1561
              S ++   Q AV  E++     +REAV     G   P  ++AP+  A + LY+KF+E+ +
Sbjct  4605  QSSLQYAWQYAVAIEVY----NIREAVRNYCPGDRLPFTLFAPNFNAARHLYAKFIEMCV  4772

Query  1562  YISIYHEGGNARDAIDYCRDFATNILSTSIDDFNVMTVIIKDDFE--QLMIRLGREGKKF  1619
             +  IY     A+       R+ +  I +   + F      ++    E   LM   G
Sbjct  4773  W--IYGVQFKAKKIKKAGREISRAIHTQ--ESFLACRAVVSRALEGGSLMFIKGMGAANA  4940

Query  1620  INIEKFPLQHPWIDRLHELA-RPVPLSAISPNISDPEQYPPLSQ--HTLFIVHPGSSFVL  1676
             +++   +    PWIDR+ ++  R  P   +SP+      + PP ++      FIV      V
Sbjct  4941  LSVPLYNTNDPWIDRIRDVKFRSAP--RMSPS-GLKMRRPPYTRVPDITFIVSENEVMVP  5111

Query  1677  ADNCTC----PPIERRKLLHSECYFAQSGLLSKSHQKFVDIVWSLRLDCEGVACLGSSTG  1732
             A C C    P I  R     E ++ +SG L   +   +D V    L    V L    G
Sbjct  5112  ASECKCHMSLPNITDR----IEGFYLRSGTLRVDNHIILDTVNRYYLSILKVVVLNDKLG  5279

Query  1733  SEAYAFASMLSCRKVYVNCLHLIGDFSPHMAGSYIPAAFVTGSLSHKLVNPSAAAVASSD  1792
             S A   A         +YV      I ++SPH AG+Y+P    +      K+  P+ +A+  +
Sbjct  5280  SAAKVIAEHPLNSSIYVAPCRDITEYSPHTAGTYVPPELLDCVALDKVAMPAPSAMIGNV  5459

Query  1793  LLLTSTVNAISSHLQGTVITGLTCDAE-LPHGSEIDGVQMLLFRLIQLVQRLTETQWVIC  1851
             L  + + +   SH+       G+    E +   SE + V+ ++  +  LV+ +    W++
Sbjct  5460  LKSSGSQSLFLSHMSRDNWNGMIFLLEDIAKHSETEEVEAIVRSVTALVESMDHVSWLMI  5639

Query  1852  KVPAVCPHVVKKFCAQLAQGFQQIRALPSLFSRP---GVLLVYLVATQRNSSVSPMHPVT  1908
                  C H +    + ++  F+ +    S F++    G L+ Y    +R S V   + P
Sbjct  5640  VGGFRCKHNLVMAMSIVSSVFEVVTFNRSPFTQSCGHGALVGY----RRRSIVGVVQPGV  5807

Query  1909  SIPSVINENQDLGETLSVNYMSQISRLFQISREERVLRNMTNVTGESSIRLACHNYIPGW  1968
              +  + N N+   E  S N + +I  +  +++ E+ L   +  T      +LAC  Y+PGW
Sbjct  5808  RVSRIQNSNRVKNEFKSGN-LWEIPAIAHLTKSEQTLWMQSVETRGDIFKLACDTYLPGW  5984

Query  1969  GHSASNSLNREEVRRSIELLKTVLLRLAS---DYVQVFFRT-----HEGLAMPRSSILPL  2020
             G           +V    E L   L +L+S   D + + +RT      E   + RS +L L
Sbjct  5985  GVG--------KVGCEQEDLLNELSKLSSYHRDNMGIVYRTILSARGEQDKVTRSFVLNL  6140

Query  2021  GQGVLHQRLNKIYMMVNRCELLLLLLTRGETLPINETVNMIKRWVSQSHEYQMRNRSFLLD  2080
              G  +Q LN+       RC +L  L +      + E    +++ V +S+  ++ N + L
Sbjct  6141  SGGGGYQTLNQQMDSRIRCLILYDLIKDPPRSLKEISYRLRQSVGRSYVLELENGNRLYG  6320

Query  2081  -LDVSDFTTNVSRYLFEILGGIYKEAFADVEGAPALILHHRIDNGRQLERIEDLFAQLPT  2139
                V  F +  S  L+ I+G + +     V  +   + H RI     + R  +L  +L
Sbjct  6321  ACQVDRFYSLYSGALYCIMGHVIQHIGLGVNSSDQALEHFRIKKP-GVHRAAELPLELEL  6497
```

```
Query  2140  RGSRPRANNSWRWNRRSEVLCWILAHRLNQGPIQVI--TSIRHVLDELHSIHHCSGIQIF  2197
             +           R+  RS  +   L         Q++  T +R +     ++ +C  I++
Sbjct  6498  KPGVQTGQVDLRYAYRSRAMVLTLLLARTLLTAQLVPDTPLRGLAT---TMRNCGYIKM-  6665

Query  2198  GEGQSSAVFLIARAADELLRATML-------NATSIYVVCDLRLCPILSQYLTIQYYLKA  2250
              E     V ++    D  +RA M         ++  + +  D +    + ++    +  +A
Sbjct  6666  TEKWDKTVNVMWIPNDYNVRAVMTQLEICADDSHPVVITIDKKRAISILEHHYCDFITRA  6845

Query  2251  PWSGIVPEIVFAICSPSSGAGMS-EVVYPDTEHLVNKYVENLITFSDCQCWDCAIEQRLC  2309
             PW+G+ PE+V         G   S +  + + E  V+   E  I    C C+DCA+E+++
Sbjct  6846  PWTGVDPEMVTLRVFEKRGYLRSLKNFHINLEETVDVAFER-IDKGACSCYDCAVEEKIL  7022

Query  2310  EEEGLTHMELDRICSLREIESVKDLR  2335
             ++ G+    L+ +   R  ES +D+R

Sbjct  7023  KDLGIPGSVLNMLYDERVKESFQDVR  7100
```

Supplementary Figure 18. Mapping of Illumina sequencing reads to the GEZL01-derived sequence. **(A)** Genome coverage by reads from three different samples (female flower – blue, male flower – red, leaf – yellow). The ORFs and identified protein domains are indicated below. Light blue dots mark putative transcription stop-start sequences. Introns 1–3 were removed before read mapping. Mapping was performed with Bowtie2 (Langmead & Salzberg, 2012) (end-to-end, seed length L 32, N 0; only reads with MAPQ > 30 and with length > 80 nt are plotted). Both strands were combined for total coverage. **(B)** Positions of spliced reads mapped to the region encoding the L protein. Reads were mapped to the GEZL01-derived sequence (before removal of any introns) using HISAT2 (Kim et al., 2019) to identify splice sites; spliced reads (only) were plotted if MAPQ > 10 and read length > 70nt. Introns 1–3 are annotated.

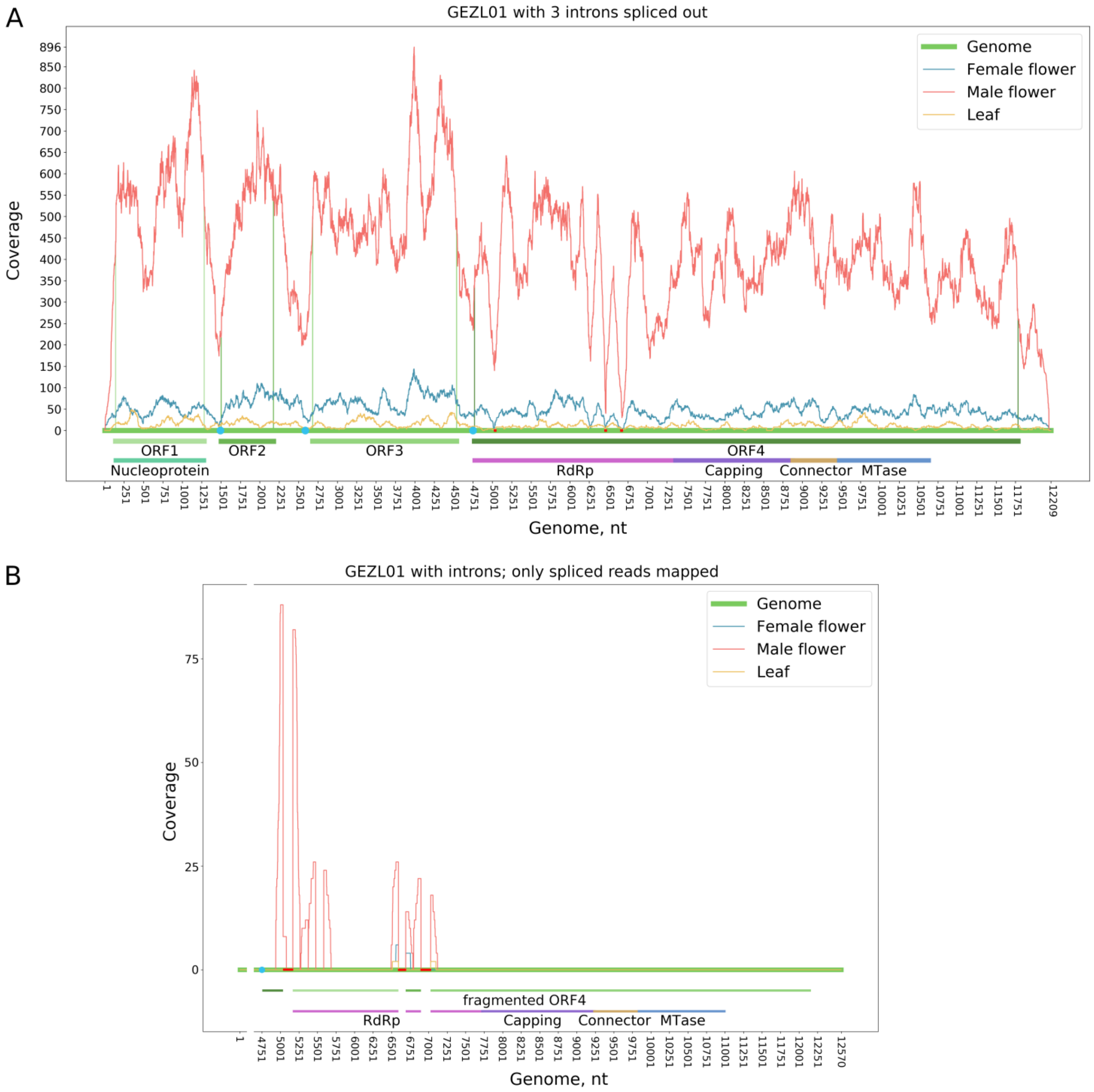

Supplementary Figure 19. Scheme for grouping HMMsearch (Eddy, 2011) results to pHMMs. Green arrows correspond to "yes", while blue arrows correspond to "no".

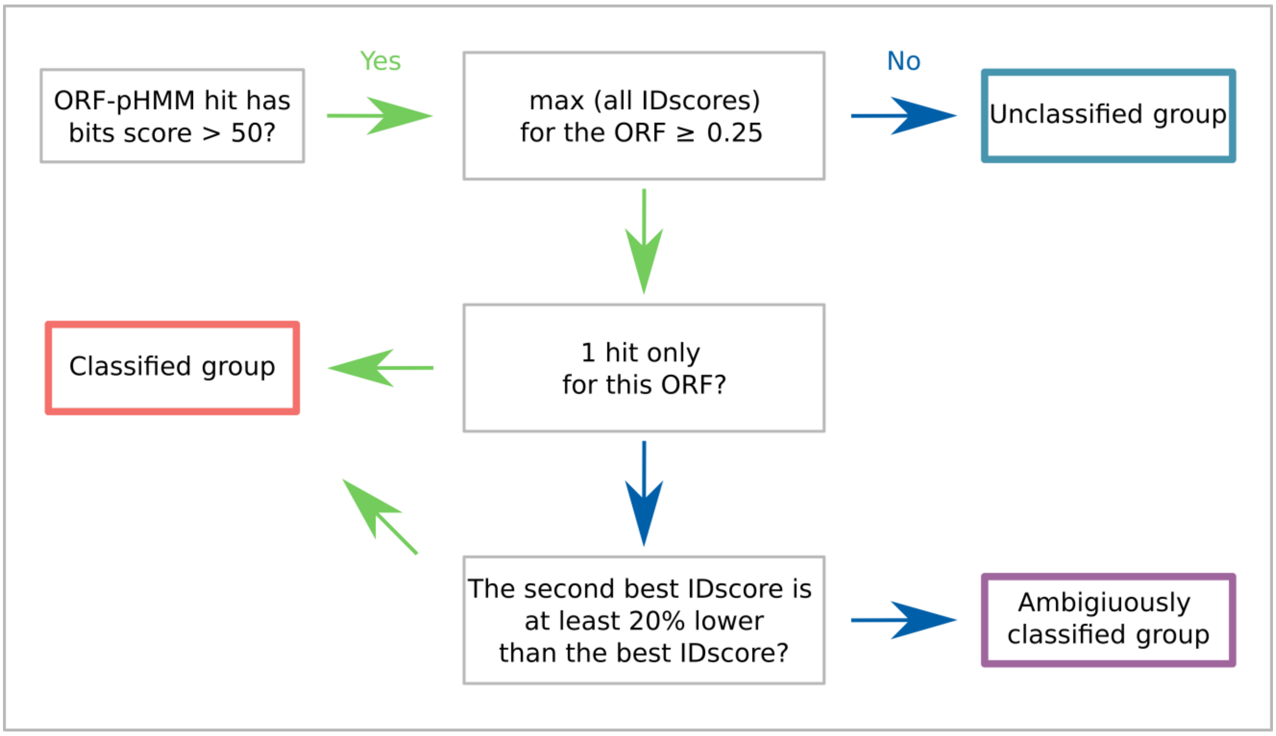

Supplementary Datasets

Supplementary Dataset 1: Excel sheet of all 15,044 putative virus-derived RdRp ORFs with associated data.

Supplementary Dataset 2: Excel sheet containing the results of our BLAST (Altschul et al., 1990; Camacho et al., 2009) and HHSearch (Steinegger et al., 2019) verification of putative viral ORFs.

Supplementary Dataset 3: PhyML (Guindon & Gascuel, 2003; Guindon et al., 2010) phylogenetic trees for the 60 clusters of classified virus-derived RdRp sequences.

Supplementary Dataset 4: The 77 viral RdRp pHMMs and their associated sequence alignments.

Supplementary Dataset 5: Alignment statistics for the input alignments for the pHMMs.

Supplementary datasets 1, 2, 3 and 5 are available at github.com/ingridole/ViralRdRp_pHMMs_2

Supplementary dataset 4 is available at github.com/ingridole/ViralRdRp_pHMMs

Supplementary Tables

Supplementary Table 1. Numbers of identified RdRp sequences, TSA datasets and unique TSA target species in different taxonomic groups. Only non-identical RdRp core sequences were used (i.e. discarding duplicate 100%-identical RdRp sequences within each classified-pHMM group, including any identical to nr/nt sequences, leaving the longest representative).

	RdRps	Datasets	Unique species	RdRps/dataset	RdRps/unique species
Plants	1440	650	275	2.22	5.24
Invertebrates	238	300	172	0.79	1.38
Arthropods	1976	918	592	2.15	3.34
Vertebrates	70	525	202	0.133	0.347
Fungi	125	77	51	1.62	2.45
Protists	60	155	110	0.387	0.545
Metagenomic	158	24	–	6.58	–
Total	4067	2649	1413	1.54	2.88

Supplementary Table 2. Influenza A virus-like sequences identified in a *Salaria pavo* TSA dataset (GEVH).

Salaria pavo TSA contig		BLASTX result (vs NCBI nr protein database); best hit when sorted by 'total score'			
Accession number	Length (nt)	Protein	Accession	Cover	Identity
GEVH01318059.1	2364	PB1	ABB04936.1	96%	99.5%
GEVH01036606.1	2362	PB2	P31345.1	96%	99.9%
GEVH01036607.1	601	PB2	ACN50227.1	49%	98.0%
GEVH01006513.1	2252	PA	Q2RF99.1	95%	100%
GEVH01127913.1	1782	HA	AFG98995.1	95%	99.8%
GEVH01391839.1	1046	M1	BAA01449.1	78%	94.1%
GEVH01289360.1	1584	NP	AAD12236.1	94%	100%
GEVH01166396.1	1490	NA	AAB03361.1	94%	99.6%
GEVH01333380.1	921	NS1	ABB04933.1	77%	100%

Supplementary Table 3. Influenza A virus-like sequences identified in a *Nibea albiflora* dataset (GFMR).

Nibea albiflora TSA contig		BLASTX result (vs NCBI nr protein database); best hit when sorted by 'total score'			
Accession number	Length (nt)	Protein	Accession	Cover	Identity
GFMR01005537.1	933	PB1	ARX92944.1	97%	99.0%
GFMR01048742.1	933	PB1	AJS16067.1	97%	99.7%
GFMR01048741.1	430	PB1	AFF27591.1	62%	98.9%
GFMR01060712.1	280	PB2	BAM76301.1	98%	98.9%
GFMR01010405.1	316	PB2	QCT26991.1	95%	99.0%
GFMR01060713.1	490	PB2	AAT65240.1	63%	99.0%
GFMR01060714.1	517	PB2	ABC48847.1	59%	97.1%
GFMR01004056.1	559	PA	ADM95244.1	74%	93.0%
GFMR01045192.1	559	PA	ADM95244.1	74%	93.0%
GFMR01002354.1	1566	NP	ABK00128.1	95%	99.6%
GFMR01040331.1	1566	NP	AAY87414.1	95%	99.0%
GFMR01008042.1	996	M1	AIX94866.1	75%	98.4%
GFMR01055396.1	996	M1	AAO52906.1	75%	99.2%
GFMR01001558.1	792	NS1	Q809Y0.1	85%	99.1%
GFMR01037854.1	792	NS1	ACA47807.1	87%	96.6%

Supplementary Table 4. Comparison of different influenza A virus strains. Proteins from influenza A virus strain A/New York/392/2004(H3N2) were queried against NCBI influenza A virus reference proteins using BLASTP (Altschul et al., 1990; Camacho et al., 2009).

Query NCBI accession	Target protein	Lowest/highest non-self cover, %	Lowest/highest non-self identity, %
YP_308849.1	PB2	100	93.9/97.6
YP_308846.1	PA	99/100	92.2/97.1
YP_308842.1	NA	98/100	43.2/85.3
YP_308843.1	NP	100	89.6/94.8
YP_308847.1	PB1	99/100	95.4/97.4
YP_308841.1	M1	100	90.9/95.2
YP_308840.1	M2	100	81.4/95.9
YP_308839.1	HA	95/100	40.2/46.7
YP_308845.1	NS1	94/100	67.0/91.3
YP_308844.1	NS2	100	81.0/97.5

Supplementary Table 5. Comparison of influenza A and influenza B virus reference sequences. Proteins from influenza A virus strain A/New York/392/2004(H3N2) were queried against NCBI influenza B virus reference proteins using BLASTP.

Query NCBI accession	Target protein	Cover, %	Identity, %
YP_308849.1	PB2	99	37.8
YP_308846.1	PA	99	36.3
YP_308842.1	NA	80	31.7
YP_308843.1	NP	96	37.8
YP_308847.1	PB1	98	61.6
YP_308841.1	M1	97	30.5
YP_308840.1	M2	none	none
YP_308839.1	HA	95	28.5
YP_308845.1	NS1	none	none
YP_308844.1	NS2	none	none

Supplementary Table 6. The Asellidae-associated clade of orthomyxovirus-like sequences.

PB1 contig accession	TSA target organism	Length (nt)	Presence in clustered tree for given identity threshold		Merged sequence details
			100%	95%	
HAEN01034382.1	*Bragasellus peltatus*	2281	+	+	
HAEX01merged	*Proasellus grafi*	2003	+	+	HAEX01038048.1 rev 936 nt + HAEX01038050.1 fwd 1092 nt
HAFI01055399.1	*Proasellus rectus*	1241	+	+	
HAFI01109142.1		386	-	-	
HAFG01101427.1	*Proasellus parvulus*	1069	+	+	
HAFG01092327.1		496	+	-	
HAEP01035468.1	*Proasellus assaforensis*	921	+	-	
HAET01043341.1	*Proasellus coxalis*	920	-	-	

Supplementary Table 7. BLASTX comparison of Asellidea-associated PB1-encoding sequences with selected *Orthomyxoviridae* NCBI reference PB1 proteins (27 July 2022). id. = identity (%), cov. = coverage (%).

**TSA organism**	**Length (nt)**	**PB1 contig accession**	**Influenza B virus**		**Dhori thogoto-virus**		**Thogoto thogoto-virus**		**Quaranfil quaranja-virus**		**Wellfleet Bay virus**		**Infectious salmon anemia virus**	
			id./ cov.	E-value	id./ cov.	E-value	id./ cov.	E-value	id./ cov.	E-value	id./ cov.	E-value	id./ cov.	E-value
Bragasellus peltatus	2281	HAEN01034382.1	28.3/91	1×10^{-53}	26.3/91	8×10^{-68}	27.9/92	2×10^{-68}	22.7/83	2×10^{-28}	22.1/83	2×10^{-31}	24.7/63	3×10^{-16}
Proasellus grafi	2003	HAEX01merged*	29.6/84	9×10^{-40}	28.0/91	3×10^{-55}	27.6/92	7×10^{-56}	24.2/59	5×10^{-16}	23.4/79	2×10^{-15}	28.7/42	2×10^{-12}
Proasellus rectus	1241	HAFI01055399.1	-	-	27.2/98	5×10^{-33}	28.3/97	3×10^{-38}	23.7/84	1×10^{-12}	24.1/51	1×10^{-10}	-	-
	386	HAFI01109142.1	-	-	-	-	-	-	-	-	-	-	-	-
Proasellus parvulus	1069	HAFG01101427.1	29.6/82	1×10^{-30}	28.3/86	2×10^{-26}	26.9/83	9×10^{-26}	23.2/81	9×10^{-13}	25.3/80	6×10^{-18}	28.6/31	1×10^{-6}
	496	HAFG01092327.1	-	-	31.1/91	4×10^{-15}	29.8/91	1×10^{-15}	-	-	-	-	-	-
Proasellus assaforensis	921	HAEP01035468.1	-	-	29.6/95	8×10^{-29}	29.8/93	5×10^{-30}	23.4/94	2×10^{-12}	28.3/70	1×10^{-10}	-	-
Proasellus coxalis	920	HAET01043341.1	-	-	29.9/95	8×10^{-29}	29.8/93	6×10^{-30}	23.4/94	3×10^{-12}	24.1/70	2×10^{-10}	-	-

* HAEX01038048.1 rev 936 nt + HAEX01038050.1 fwd 1092 nt

Supplementary Table 8. Divergent orthomyxovirus-like TSA and nr/nt sequences. All contigs were compared using BLASTX against the entire nr protein NCBI database (Sayers et al., 2022; 17 July 2020) and the best scoring matches are shown. id. = identity (%), cov. = coverage (%).

Orthomyxovirus-like sequence				BLASTX best nr match			
Data-base	Accession number	Target organism (TSA) or virus name (nr/nt)	Contig length (nt)	Protein accession	Virus name	E-value	id./ cov.
TSA	GFBM010604515.1	*Ambystoma mexicanum*	2551	QKK82921.1 (PB1)	Neke Harbour virus	2×10^{-8}	24.6/33
TSA	JP343076.1	*Lepeophtheirus salmonis*	557	MG600033.1 (PB1)	Wenling orthomyxo-like virus 1	3×10^{-10}	85.3/12
TSA	GFRZ01merged (GFRZ01104574.1 + GFRZ01104573 .1)	*Symsagittifera roscoffensis*	1823 (1262 + 605)	AVM87616.1 (PB1)	Wenling orthomyxo-like virus 1	9×10^{-40}	32.8/73
TSA	GFAT01merged (GFAT01052308.1 + GFAT01014782.1rv)	*Mnemiopsis leidyi*	2452 (1659 + 1987)	ASM94088.1 (PB1)	Barns Ness dog whelk orthomyxo-like virus 1	2×10^{-10}	25.9/54
TSA	GFAT01139990.1	*Mnemiopsis leidyi*	723	AVM87618.1 (PB1)	Yancheng orthomyxo-like virus	5×10^{-6}	28.7/94
TSA	GFKU01079218.1	*Apostichopus japonicus*	2170	APG77905.1 (PB1)	Hubei orthomyxo-like virus 5	5×10^{-9}	20.9/81
				**Best non-self BLASTX nr match**			
nr/nt	MG600033.1	Wenling orthomyxo-like virus 1	2324	ASM94088.1 (PB1)	Barns Ness dog whelk orthomyxo-like virus 1	2×10^{-83}	32.0/90
nr/nt	MF190045.1	Barns Ness dog whelk orthomyxo-like virus 1	2219	AVM87618.1 (PB1)	Yancheng orthomyxo-like virus	2×10^{-94}	34.3/86
nr/nt	MG600035.1	Yancheng orthomyxo-like virus	2055	ASM94088.1 (PB1)	Barns Ness dog whelk orthomyxo-like virus 1	1×10^{-94}	34.3/92
nr/nt	NC_033319.1	Changping earthworm virus 2	4027	AVM87618.1 (PB1)	Yancheng orthomyxo-like virus	0.19	24.8/22

Supplementary Table 9. List of RdRp pHMMs with the corresponding number of input sequences, RdRp cropping coordinates, and the HMMbuild (Eddy, 2011) output information.

Taxonomic group	pHMM profile name	Number of sequences	Consensus alignment length, aa	RdRp pHMM used for cropping (2nd best hit or core of best hit)	Cropping coordinates of an alignment (from start: from end)	pHMM length, aa	Effective number of sequences	Relative entropy per position
dsRNA	Birnaviridae	8	575	RdRP_1	185:-343	506	0.77	0.592
	Chrysoviridae	7	602	RdRP_4	313:-207	598	0.65	0.592
	Giardiavirus	1	461	RdRP_4	240:-356	461	1	0.567
	Megabirnaviridae	3	486	RdRP_1	365:-304	482	0.49	0.593
	Partitiviridae	67	616	RdRP_1	196:-115	474	3.35	0.59
	Picobirnaviridae	5	544	RdRP_4	0:-0	533	0.55	0.591
	Quadriviridae	1	506	RdRP_4	559:-245	506	1	0.565
	Sedoreovirinae_1	29	716	RdRP_4	420:-592	601	2.79	0.59
	Sedoreovirinae_2	8	644	RdRP_4	147:-410	603	0.88	0.591
	Spinareovirinae_1	6	707	1N35_A PDB	679:-531	706	0.59	0.591
	Spinareovirinae_2	15	426	RdRP_5	77:-41	363	2.27	0.59
	Totiviridae	10	503	RdRP_4	863:-225	473	1.35	0.59
+ssRNA	Alphaflexiviridae	52	474	RdRP_2	1806:-109	434	0.98	0.591
	Alphatetraviridae	2	433	RdRP_2	931:-348	433	0.44	0.592
	Arteriviridae	14	454	RdRP_1	2975:-910	447	0.77	0.59
	Astroviridae	11	482	RdRP_1	1171:-6	463	0.7	0.589
	Barnaviridae	1	420	RdRP_1	0:-0	420	1	0.6
	Betaflexiviridae	87	436	RdRP_2	2511:-754	421	0.89	0.589
	Bromoviridae	28	470	RdRP_2	480:-176	440	1.19	0.59
	Caliciviridae	28	518	RdRP_1	1632:-635	482	1.68	0.59
	Chipolycivirus	2	510	RdRP_1	2026:-33	507	0.53	0.587
	Closteroviridae	20	451	RdRP_2	56:-115	446	1.16	0.59
	Coronaviridae	22	497	RdRP_1	5440:-1844	497	0.48	0.589
	Deltaflexiviridae	2	447	RdRP_2	1655:-0	447	0.43	0.589
	Dicistroviridae	14	582	RdRP_1	1721:-0	525	1.21	0.591
	Endornaviridae	24	502	RdRP_2	6553:-43	483	1.7	0.59
	Flaviviridae	138	541	RdRP_3	5231:-188	431	2.97	0.59
	Gammaflexiviridae	1	422	RdRP_2	1453:-21	422	1	0.575
	Hepeviridae	5	436	RdRP_2	1380:-9	430	0.7	0.588
	Hypoviridae	14	602	Flavi_RdRP	3272:-1464	454	1.79	0.59
	Iflaviridae	27	570	RdRP_1	3765:-175	524	1.66	0.59
	Iflaviridae_4	4	485	RdRP_1	2608:-46	485	0.46	0.586
	Luteoviridae	3	480	RdRP_1	871:-0	479	0.47	0.587
	Marnaviridae	1	496	RdRP_1	1196:-889	496	1	0.577
	Mesoniviridae	9	513	RdRP_2	3067:-1580	512	0.45	0.593
	Narnaviridae	40	600	RdRP_1	240:-370	394	4.02	0.59
	Nodaviridae	3	497	RdRP_3	376:-162	492	0.58	0.589
	Permutotetraviridae	1	486	RdRP_1	120:-651	486	1	0.583
	Picornaviridae	138	603	RdRP_1	4310:-122	462	2.44	0.59
	Polycipiviridae	11	531	RdRP_1	2086:-27	516	0.89	0.589
	Potyviridae	186	515	RdRP_1	3749:-868	469	1.65	0.591
	Roniviridae	1	523	RdRP_1	4517:-1633	523	1	0.578
	Rubellavirus	1	439	RdRP_2	355:-21	439	1	0.573
	Secoviridae	97	734	RdRP_1	3483:-484	494	14.16	0.59
	Sobemoviridae	12	477	RdRP_1	3877:-120	461	0.71	0.59
	Solinviviridae	2	585	RdRP_1	2040:-1098	584	0.57	0.59
	Togaviridae	32	477	RdRP_2	2233:-6	475	1.17	0.589
	Tombusviridae	49	566	RdRP_3	728:-141	506	1.33	0.59
	Tymoviridae	36	474	RdRP_2	1926:-420	422	0.68	0.588
	Virgaviridae	30	489	RdRP_2	1260:-7	473	0.63	0.592
−ssRNA	Artoviridae	7	479	RdRP_3	347:-1506	479	0.8	0.589
	Bornaviridae	13	528	Arena_RNA_pol	379:-833	527	0.45	0.587
	Chuviridae	29	516	Arena_RNA_pol	614:-1731	478	1.46	0.589
	Cruliviridae	1	744	Bunya_RdRp	581:-848	744	1	0.566
	Filoviridae	9	423	Arena_RNA_pol	542:-1387	423	0.46	0.587
	Fimoviridae	9	485	Arena_RNA_pol	1029:-859	472	0.61	0.592
	Hantaviridae	30	742	Bunya_RdRp	559:-873	740	0.48	0.591
	Mymonaviridae	6	478	Arena_RNA_pol	569:-1123	461	0.98	0.591
	Mypoviridae	1	539	Bunya_RdRp	540:-58	539	1	0.576
	Nairoviridae	14	543	Arena_RNA_pol	2672:-1628	528	0.77	0.588
	Nyamiviridae	5	490	Arena_RNA_pol	493:-1085	486	0.66	0.593
	Ophioviridae	4	424	Arena_RNA_pol	554:-1542	421	0.54	0.591
	Orthomyxoviridae	7	518	Arena_RNA_pol	250:-69	473	1.32	0.591
	Paramyxoviridae	60	724	Arena_RNA_pol	704:-1304	566	0.91	0.59
	Peribunyaviridae	36	638	Arena_RNA_pol	1186:-1042	465	0.76	0.589

	Phasmaviridae	8	795	Bunya_RdRp	672:-1233	670	1.11	0.591
	Phenuiviridae	14	560	Bunya_RdRp	1444:-1323	522	1.27	0.589
	Pneumoviridae	7	434	Arena_RNA_pol	577:-1171	433	0.46	0.589
	Qinviridae	8	457	Arena_RNA_pol	632:-1081	421	1.25	0.589
	Rhabdoviridae	153	462	RdRP_3	660:-1900	400	1.8	0.589
	Tospoviridae	17	410	Arena_RNA_pol	1291:-1286	404	0.48	0.586
	Wupedeviridae	1	514	Arena_RNA_pol	2038:-1150	514	1	0.55
	Xinmoviridae	5	416	Arena_RNA_pol	564:-1278	411	0.79	0.59
	Yueviridae	2	426	Bunya_RdRp	503:-1258	426	0.49	0.586
+ssRNA or dsRNA	Yanvirus	3	503	RdRP_2	840:-47	465	0.74	0.591
	Zhaovirus	9	581	RdRP_2	2231:-609	440	2.15	0.589
	Weivirus	18	559	RdRP_3	496:-129	434	1.65	0.59

Supplementary Table 10. Search settings used to query the NCBI non-redundant nucleotide database (Sayers et al., 2022).

Group	Number of sequences	Query used to retrieve sequences	Download date
dsRNA viruses	48,989	txid35325[Organism:exp] AND (viruses[filter] AND ("1000"[SLEN] : "1000000"[SLEN]))	14 May 2018
−ssRNA viruses excluding *influenza A virus*	123,576	txid35301[Organism:exp] AND (viruses[filter] AND ("1000"[SLEN] : "1000000"[SLEN])) NOT txid11320[Organism:exp]	14 May 2018
+ssRNA viruses excluding *hepacivirus C virus*	91,797	txid35278[Organism:exp] AND (viruses[filter] AND ("1000"[SLEN] : "1000000"[SLEN])) NOT txid11103[Organism:exp]	15 May 2018
unassigned ssRNA viruses	5	txid674978[Organism:exp] AND (viruses[filter] AND ("1000"[SLEN] : "1000000"[SLEN]))	14 May 2018
unclassified ssRNA viruses	59	txid439490[Organism:exp] AND (viruses[filter] AND ("1000"[SLEN] : "1000000"[SLEN]))	14 May 2018
unassigned viruses	0	txid686617[Organism:exp]	14 May 2018
unclassified archaeal viruses	22	txid451344[Organism:exp] AND (viruses[filter] AND ("1000"[SLEN] : "1000000"[SLEN]))	14 May 2018
unclassified bacterial viruses	1833	txid12333[Organism:exp] AND (viruses[filter] AND ("1000"[SLEN] : "1000000"[SLEN]))	14 May 2018
unclassified RNA viruses	3216	txid1922347[Organism:exp] AND (viruses[filter] AND ("1000"[SLEN] : "1000000"[SLEN]))	14 May 2018
unclassified virophages	17	txid552364[Organism:exp]	14 May 2018
unclassified viruses	1362	txid12429[Organism:exp] AND (viruses[filter] AND ("1000"[SLEN] : "1000000"[SLEN]))	14 May 2018
environmental samples	3745	txid186616[Organism:exp] AND (viruses[filter] AND ("1000"[SLEN] : "1000000"[SLEN]))	14 May 2018

Supplementary Table 11. Numbers of sequences before and after applying CD-HIT-EST (Li & Godzik, 2006; Fu et al., 2012) to remove similar sequences.

**Group**	**Number of sequences**	
	**Before CD-HIT-EST**	**After CD-HIT-EST**
dsRNA viruses	48,989	3259
−ssRNA viruses (excluding *influenza A virus*)	123,576	2506
+ssRNA viruses (excluding *hepacivirus C virus*)	91,797	4561
Others combined	7059	4506
**Total**	**271,421**	**14,832**

Supplementary Table 12. Numbers of ORFs found for each genetic code table for each sequence dataset.

**Sequences**	**Number of ORFs**			
	**Table 1**	**Table 4**	**Table 6**	**Total**
Reference	6,618,649	5,842,373	5,257,850	**17,718,872**
nr/nt	1,105,977	1,049,250	940,742	**3,095,969**
TSA	4,888,516,023	4,484,941,615	4,219,750,493	**13,593,208,131**
**Totals**	4,896,240,649	4,491,833,238	4,225,949,085	**13,614,022,972**
**Grand total**				

Supplementary Methods

Verification of pHMM matches

In order to verify that sequences identified using the pHMMs were true viral RdRps rather than false positives, and to identify any chimeric sequences, all ORFs identified as encoding a viral RdRp or fragments thereof were checked using a BLAST-based approach. Candidate RdRp-encoding ORF sequences were compared to the NCBI non-redundant (nr) protein database, downloaded 19 Jun 2022, using the DIAMOND v.0.9.14 (Buchfink et al., 2021) implementation of BLASTP (Altschul et al., 1990; Camacho et al., 2009) with the default settings, filtered to keep only hits with an amino acid identity >30% and an alignment length >50 amino acids. Where a more sensitive search was required, sequences were compared to either the full nr database or the subset of this database derived from *Orthornavirae* using the online BLASTP web server (blast.ncbi.nlm.nih.gov; Altschul et al., 1990; Camacho et al., 2009; searches performed 10–17 Aug 2022), using the default word size of six initially and a word size of three where required. Online BLASTP hits were only filtered to have an E-value < 0.05.

BLAST target sequences were classified as known RNA viruses, uncharacterised proteins or non-viral proteins. For the uncharacterised proteins (proteins with names including uncharacterised, unclassified, unnamed, hypothetical or similar), many of which are likely to be RNA viral in origin, we used the DIAMOND settings above to verify similarity to at least one known RNA virus; sequences meeting these criteria are referred to hereafter as uncharactered virus-like proteins (UVPs). RNA viral sequences without an NCBI taxonomic classification were also assigned as *Orthornavirae* in this manner. Sequences were considered to be valid and non-chimeric if they met the following criteria: (i) the best match against any sequence was a significant (E-value <0.05) hit against an *Orthornavirae* sequence or a UVP, (ii) there was at least one significant match against an *Orthornavirae* sequence, and (iii) if the best hit was not an *Orthornavirae* sequence then all hits scoring higher than the best *Orthornavirae* hit were UVPs. Chimeric sequences were those which met these criteria except that not all sequences scoring higher than the best scoring *Orthornavirae* sequence were UVPs. Using this method, all 12,136 ORFs were valid hits, and 25 of these were chimeric.

For verification with an alternative HMM-based approach, HHSearch (part of HHSuite v3.3.0; Steinegger et al., 2019) was used on our set of putative viral ORFs, searching against the full Pfam database (Finn et al., 2014; version 35), with the default settings. Results were then filtered to include only those with a query length >20, probability >20 and p-value <0.05. Where no RdRp was detected, inferred alignments were generated using HHBlits (also part of HHSuite) searching against the uniclust30_2018_08 database (Mirdita et al., 2017) with one iteration. These were then used as queries against the same Pfam database, first with HHBlits, then – if no RdRp was detected – with HHSearch (which is more sensitive). Pfam results were manually classified as RNA viral RdRp, RNA viral non-RdRp, non-RNA-viral, unknown (based on annotation as “domain of unknown function” or similar) or uninformative, a category consisting of helicase and AAA domains which are common in both RNA viruses and cellular proteins (Hickman & Dyda, 2005). Sequences were considered to be valid if either the best match was a viral RdRp or everything scoring higher than the best scoring viral RdRp was a viral non-RdRp, unknown or uninformative.

To compare BLASTP directly with HMMSearch, the 1,784 cropped RdRp sequences used to create the 77 viral pHMMs were used to create a BLASTP (Altschul et al., 1990; Camacho et al., 2009;

standalone version 2.12.0) database and this database was searched using the default settings with the 12,136 RdRp-encoding ORF sequences as queries. All of our viral ORFs were detected, using a relaxed E-value cutoff of 0.05. To establish the false positive rate, a reference set of human proteins, the “reviewed, Swiss-Prot” set, which should not be RNA viral in origin, was downloaded from UniprotKB (UniProt Consortium, 2021; downloaded from https://www.uniprot.org on 26 Aug 2022) and compared to the same database with the same settings; here, 824 significant hits were found. HMMSearch was applied to the same set of proteins using the settings decribed above and our 77 pHMM profiles; here, no results were detected with a p-value (adjusted for database size) meeting our criteria.

For the comparison with pHMMs from the SUPERFAMILY database (Gough et al. 2001), 11 pHMMs were extracted from the SUPERFAMILY Hidden Markov Model set, version 1.75. These were the pHMMs with model IDs of 44175, 46068, 47809, 49138, 49312, 49395, 51076, 51077, 51078, 54504. These are all from SCOP superfamily 56672, DNA/RNA polymerases and are all classified with “Family of seed” as RNA-dependent RNA-polymerase. Our ORFs were compared to these pHMMs with HMMsearch, with settings as described in the main text for our earlier HMMsearch analyses.

Supplementary References

Altschul, S. F., Gish, W., Miller, W., Myers, E. W., & Lipman, D. J. (1990). ‘Basic local alignment search tool’, *Journal of Molecular Biology*, 215/3: 403–10.

Brown, J. W., Smith, P., & Simpson, C. G. (1996). ‘Arabidopsis consensus intron sequences’, *Plant Molecular Biology*, 32/3: 531–5.

Buchfink, B., Reuter, K., & Drost, H.-G. (2021). ‘Sensitive protein alignments at tree-of-life scale using DIAMOND’, *Nature Methods*, 18/4: 366–8.

Camacho, C., Coulouris, G., Avagyan, V., Ma, N., Papadopoulos, J., Bealer, K., & Madden, T. L. (2009). ‘BLAST+: architecture and applications’, *BMC bioinformatics*, 10: 421.

Cock, P. J. A., Antao, T., Chang, J. T., Chapman, B. A., Cox, C. J., Dalke, A., Friedberg, I., et al. (2009). ‘Biopython: freely available Python tools for computational molecular biology and bioinformatics’, *Bioinformatics*, 25/11: 1422–3.

Crooks, G. E., Hon, G., Chandonia, J.-M., & Brenner, S. E. (2004). ‘WebLogo: a sequence logo generator’, *Genome Research*, 14/6: 1188–90.

Eddy, S. R. (2011). ‘Accelerated Profile HMM Searches’, *PLoS computational biology*, 7/10: e1002195.

Edgar, R. C. (2004). ‘MUSCLE: a multiple sequence alignment method with reduced time and space complexity’, *BMC Bioinformatics*, 5/1: 113.

Finn, R. D., Bateman, A., Clements, J., Coggill, P., Eberhardt, R. Y., Eddy, S. R., Heger, A., et al. (2014). ‘Pfam: the protein families database’, *Nucleic Acids Research*, 42/Database issue: D222-230.

Fu, L., Niu, B., Zhu, Z., Wu, S., & Li, W. (2012). ‘CD-HIT: accelerated for clustering the next-generation sequencing data’, *Bioinformatics (Oxford, England)*, 28/23: 3150–2.

Gough, J. Karplus, K., Hughey, R., Chothia, C. (2001) Assignment of homology to genome sequences using a library of hidden Markov models that represent all proteins of known structure. *Journal of Molecular Virology*. 313(4):903-19

Guindon, S., Dufayard, J.-F., Lefort, V., Anisimova, M., Hordijk, W., & Gascuel, O. (2010). ‘New algorithms and methods to estimate maximum-likelihood phylogenies: assessing the performance of PhyML 3.0’, *Systematic Biology*, 59/3: 307–21.

Guindon, S., & Gascuel, O. (2003). ‘A simple, fast, and accurate algorithm to estimate large phylogenies by maximum likelihood’, *Systematic Biology*, 52/5: 696–704.

Hagberg, A., Swart, P., & S Chult, D. (2008). ‘Exploring network structure, dynamics, and function using networkx’, *Proceedings of the 7th Python in Science Conference (SciPy)*, 11–5.

Hickman, A. B., & Dyda, F. (2005). ‘Binding and unwinding: SF3 viral helicases’, *Current Opinion in Structural Biology*, 15/1: 77–85.

Kim, D., Paggi, J. M., Park, C., Bennett, C., & Salzberg, S. L. (2019). ‘Graph-based genome alignment and genotyping with HISAT2 and HISAT-genotype’, *Nature Biotechnology*, 37/8: 907–15.

Langmead, B., & Salzberg, S. L. (2012). ‘Fast gapped-read alignment with Bowtie 2’, *Nature Methods*, 9/4: 357–9.

Li, W., & Godzik, A. (2006). ‘Cd-hit: a fast program for clustering and comparing large sets of protein or nucleotide sequences’, *Bioinformatics (Oxford, England)*, 22/13: 1658–9.

Mirdita, M., von den Driesch, L., Galiez, C., Martin, M. J., Söding, J., & Steinegger, M. (2017). ‘Uniclust databases of clustered and deeply annotated protein sequences and alignments’, *Nucleic Acids Research*, 45/D1: D170–6.

Sayers, E. W., Bolton, E. E., Brister, J. R., Canese, K., Chan, J., Comeau, D. C., Connor, R., et al. (2022). ‘Database resources of the national center for biotechnology information’, *Nucleic Acids Research*, 50/D1: D20–6.

Shannon, P., Markiel, A., Ozier, O., Baliga, N. S., Wang, J. T., Ramage, D., Amin, N., et al. (2003). ‘Cytoscape: a software environment for integrated models of biomolecular interaction networks’, *Genome Research*, 13/11: 2498–504.

Steinegger, M., Meier, M., Mirdita, M., Vöhringer, H., Haunsberger, S. J., & Söding, J. (2019). ‘HH-suite3 for fast remote homology detection and deep protein annotation’, *BMC bioinformatics*, 20/1: 473.

UniProt Consortium. (2021). ‘UniProt: the universal protein knowledgebase in 2021’, *Nucleic Acids Research*, 49/D1: D480–9.

Wolf, Y. I., Kazlauskas, D., Iranzo, J., Lucía-Sanz, A., Kuhn, J. H., Krupovic, M., Dolja, V. V., et al. (2018). ‘Origins and Evolution of the Global RNA Virome’, *mBio*, 9/6: e02329-18.

Wolf, Y. I., Silas, S., Wang, Y., Wu, S., Bocek, M., Kazlauskas, D., Krupovic, M., et al. (2020). ‘Doubling of the known set of RNA viruses by metagenomic analysis of an aquatic virome’, *Nature Microbiology*, 5/10: 1262–70.
